# A global invasion by the thrip, *Frankliniella occidentalis*: Current virus vector status and its management

**DOI:** 10.1111/1744-7917.12721

**Published:** 2019-10-23

**Authors:** Zhen He, Jing‐Fei Guo, Stuart R. Reitz, Zhong‐Ren Lei, Sheng‐Yong Wu

**Affiliations:** ^1^ State Key Laboratory for Biology of Plant Diseases and Insect Pests, Institute of Plant Protection Chinese Academy of Agricultural Sciences Beijing China; ^2^ School of Horticulture and Plant Protection Yangzhou University Yangzhou Jiangsu Province China; ^3^ Malheur Experiment Station Oregon State University Ontario OR USA

**Keywords:** global distribution, integrated pest management, invasion, thrips, viruses transmission

## Abstract

Western flower thrip, *Frankliniella occidentalis* (Pergande), is among the most economically important agricultural pests globally, attacking a wide range of vegetable and horticultural crops. In addition to causing extensive crop damage, the species is notorious for vectoring destructive plant viruses, mainly belonging to the genera *Orthotospovirus*, *Ilarvirus*, *Alphacarmovirus* and *Machlomovirus*. Once infected by orthotospoviruses, thrips can remain virulent throughout their lifespan and continue transmitting viruses to host plants when and wherever they feed. These irruptive viral outbreaks in crops will permanently disrupt functional integrated pest management systems, and typically require a remedial treatment involving insecticides, contributing to further development of insecticide resistance. To mitigate against this continuing cycle, the most effective management is early and comprehensive surveillance of the pest species and recognition of plant viruses in the field. This review provides information on the pest status of *F. occidentalis*, discusses the current global status of the viruses vectored by this thrip species, examines the mechanisms involved in transmitting virus‐induced diseases by thrips, and reviews different management strategies, highlighting the potential management tactics developed for various cropping systems. The early surveillance and the utilization of potential methods for control of both *F. occidentalis* and viruses are proposed.

## Introduction

**Figure 1:**
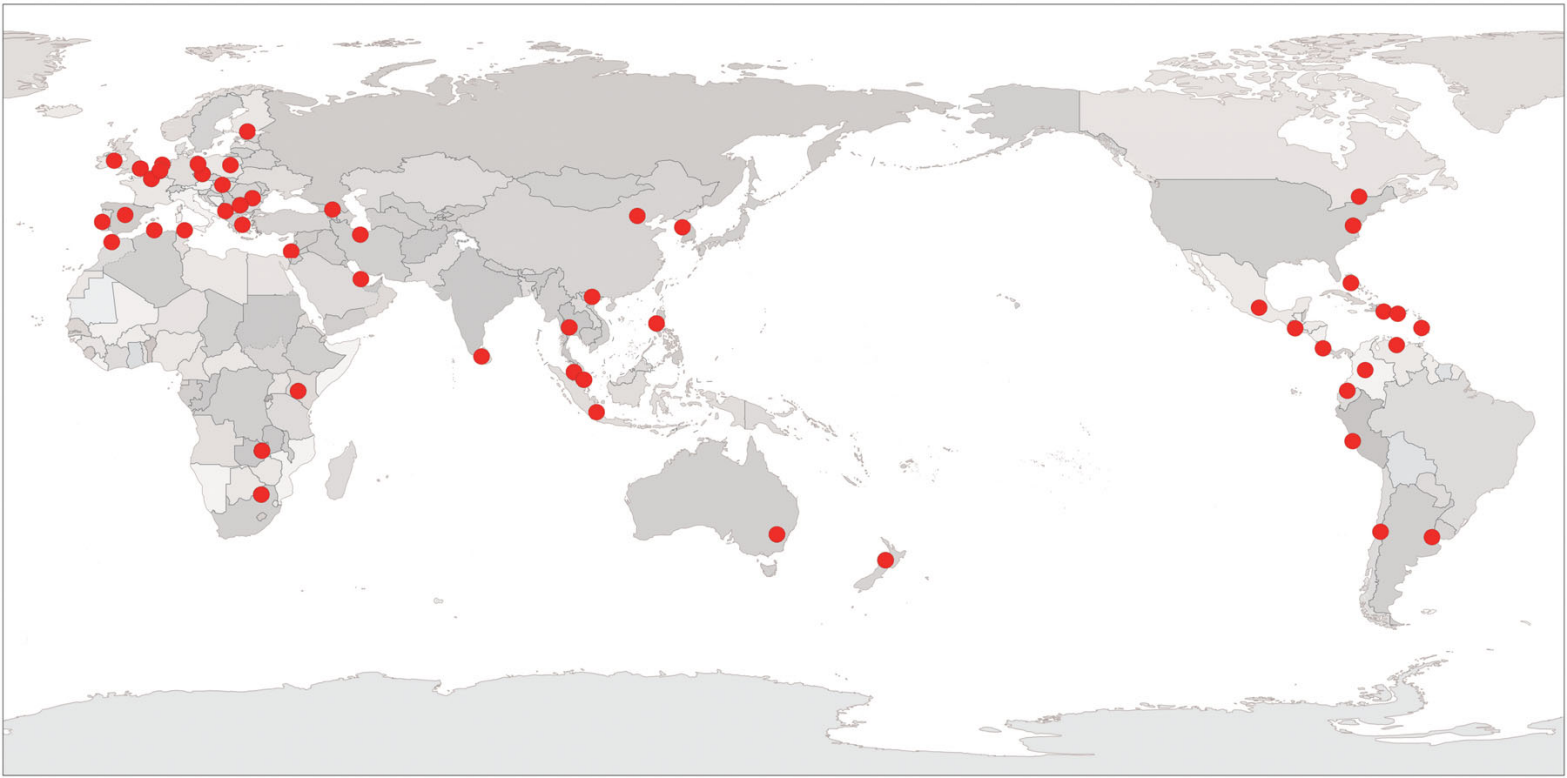
The worldwide distribution of *Frankliniella occidentalis*. GS (2019) 4551.

Thrips (order Thysanoptera) are minute insects only a few millimeters or less in length. Of the approximately 5500 described species of thrips in the world, scarcely 1% are considered to be serious pests of commercial crops (Morse & Hoddle, 2006; Healey *et al*., 2017). Among these pests, several species stand out as being among the most important global agricultural pests. These include four of the major thrip pests, the western flower thrip, *Frankliniella occidentalis* Pergande, the onion thrip, *Thrips tabaci* Lindeman, the melon thrip, *T. palmi* Karny and the yellow tea thrip (chili thrip), *Scirtothrips dorsalis* Hood (Mound, 2002; Riley *et al*., 2018). *F. occidentalis* is a polyphagous and ubiquitous invader of key agri‐ and horticultural crops in diverse field and greenhouse environments. This is due to the damage caused directly by its feeding and oviposition, and indirectly through transmission of plant viruses, of which tomato spotted wilt orthotospovirus (TSWV) is the most economically important (Schneweis *et al*., 2017). *F. occidentalis* was first described in 1895 in California, USA, and beginning in the late 1970s has since become a major global pest (Kirk & Terry, 2003). This species has been the most intensively studied member of the order Thysanoptera since 1980, accounting for over one‐third of the publications based on this order (Reitz *et al*., 2011). *F. occidentalis* has continued its spread around the world and is now distributing in at least 57 countries (Fig. 1, Table S1). The spread of *F. occidentalis* and various vectored orthotospoviruses have frequently caused failure of established integrated pest management (IPM) systems for agricultural crops (Morse & Hoddle, 2006).

Phytophagous thrips have many traits that predispose them to be successful invaders, such as minute size, cryptic habits, high reproductive potential and high dispersal capability. *F. occidentalis* has superior or additional features, promoting their worldwide spread and sustained damage. The possible reasons for the start of the spread is intensive insecticide use in the 1970s and 1980s, which was reviewed by Kirk and Terry (2003). Biological factors facilitating invasion by thrips was reviewed by Morse and Hoddle (2006). Biological processes and molecular interactions involved in the virus acquisition and transmission by thrips was reviewed by Whitfield *et al*. (2005a). Over a decade after these reports, we aim to provide a summary of the extraordinary attributes that make for a successful invader with major economical damage potential. We have reconstructed in a chronological order the current global distribution of *F. occidentalis*, as well as several viruses transmitted. We have also summarized the control strategies based on IPM of *F. occidentalis*, stressing the recent progress in biological control.

## Biology and ecology

*F. occidentalis* possesses several biological and ecological characteristics that enable it to become a dominant thrip species in many of the areas it has invaded. Its short generation time and high reproductive potential, often with a predisposition to parthenogenesis, enhances the likelihood of establishment (Kirk & Terry, 2003; Zhang *et al*., 2010); its cryptic behavior and high level of vagility enable it to disperse to a wide variety of crops (Cloyd, 2009; Reitz, 2009); its polyphagous nature likely supplements its predisposition to evolve resistance to many classes of insecticides through metabolic detoxification pathways (Demirozer *et al*., 2012); its widespread resistance to most major insecticides, in turn, makes it difficult to control (Bielza, 2010; Gao *et al*., 2012a); its highly efficient exploitation of food sources provides it with a competitive advantage over indigenous species and enables it to become successfully established in new regions (Morse & Hoddle, 2006; Demirozer *et al*., 2012). However, its propensity to transmit viruses often results in serious losses in a wide range of crops (Wijkamp *et al*., 2010; Ogada & Poehling, 2015). In most cases, combinations of these attributes contribute to its high invasion success, ultimately resulting in severe economic damage to crops throughout the world.

## Viruses transmitted by *F. occidentalis*

**Figure 2:**
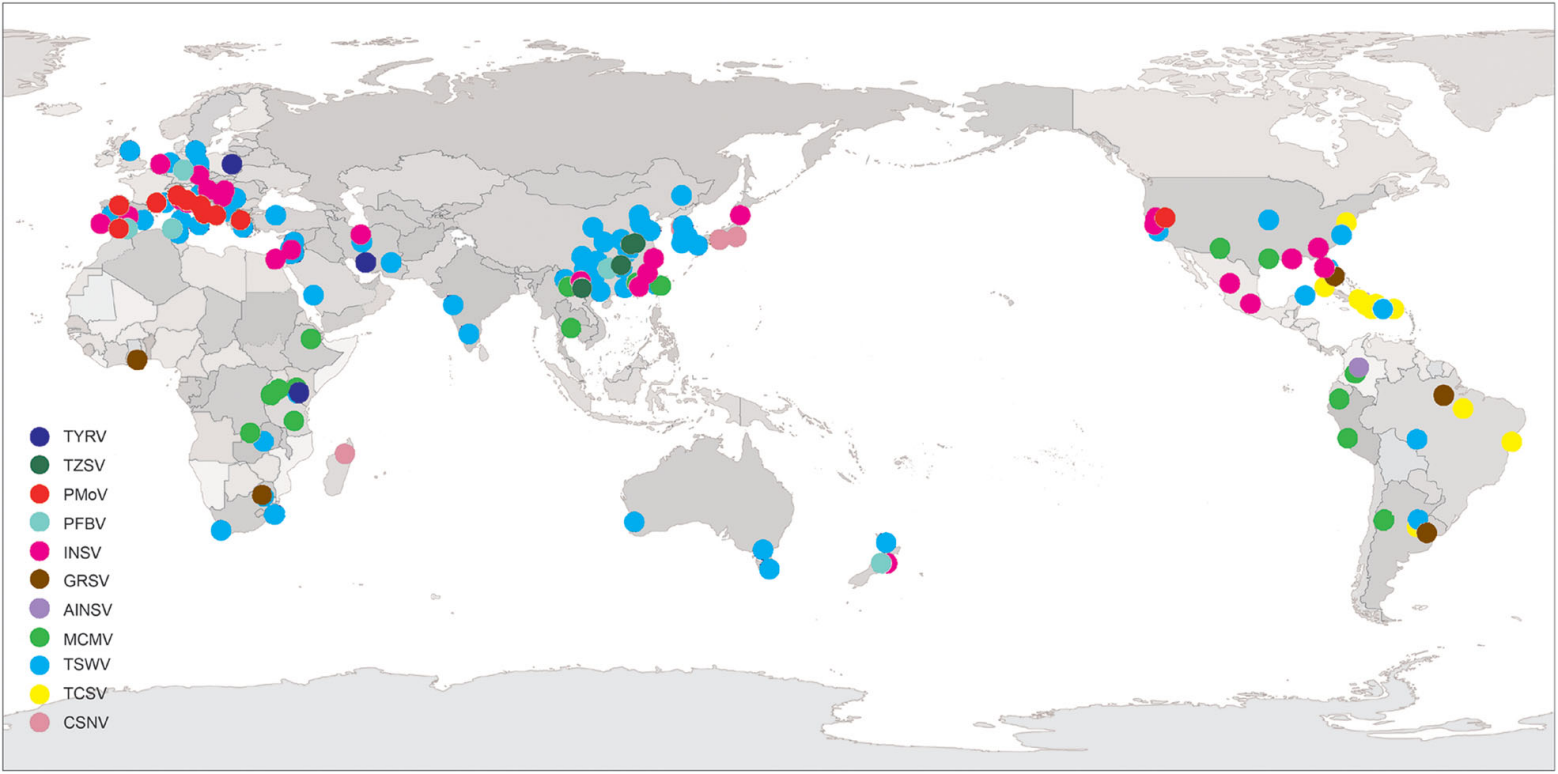
The worldwide distribution of viruses transmitted by *Frankliniella occidentalis*. The viruses shown in the map are: AlNSV, alstroemeria necrotic streak orthotospovirus; CSNV, chrysanthemum stem necrosis orthotospovirus; GRSV, groundnut ringspot orthotospovirus; INSV, impatiens necrotic spot orthotospovirus; TCSV, tomato chlorotic spot orthotospovirus; TSWV, tomato spotted wilt orthotospovirus; TYRV, tomato yellow ring virus; TZSV, tomato zonate spot orthotospovirus; PMoV, parietaria mottle virus; PFBV, pelargonium flower break virus; MCMV, maize chlorotic mottle virus. Arcgis 10.0 software was used to create the map. GS (2019) 4551.

**Figure 3:**
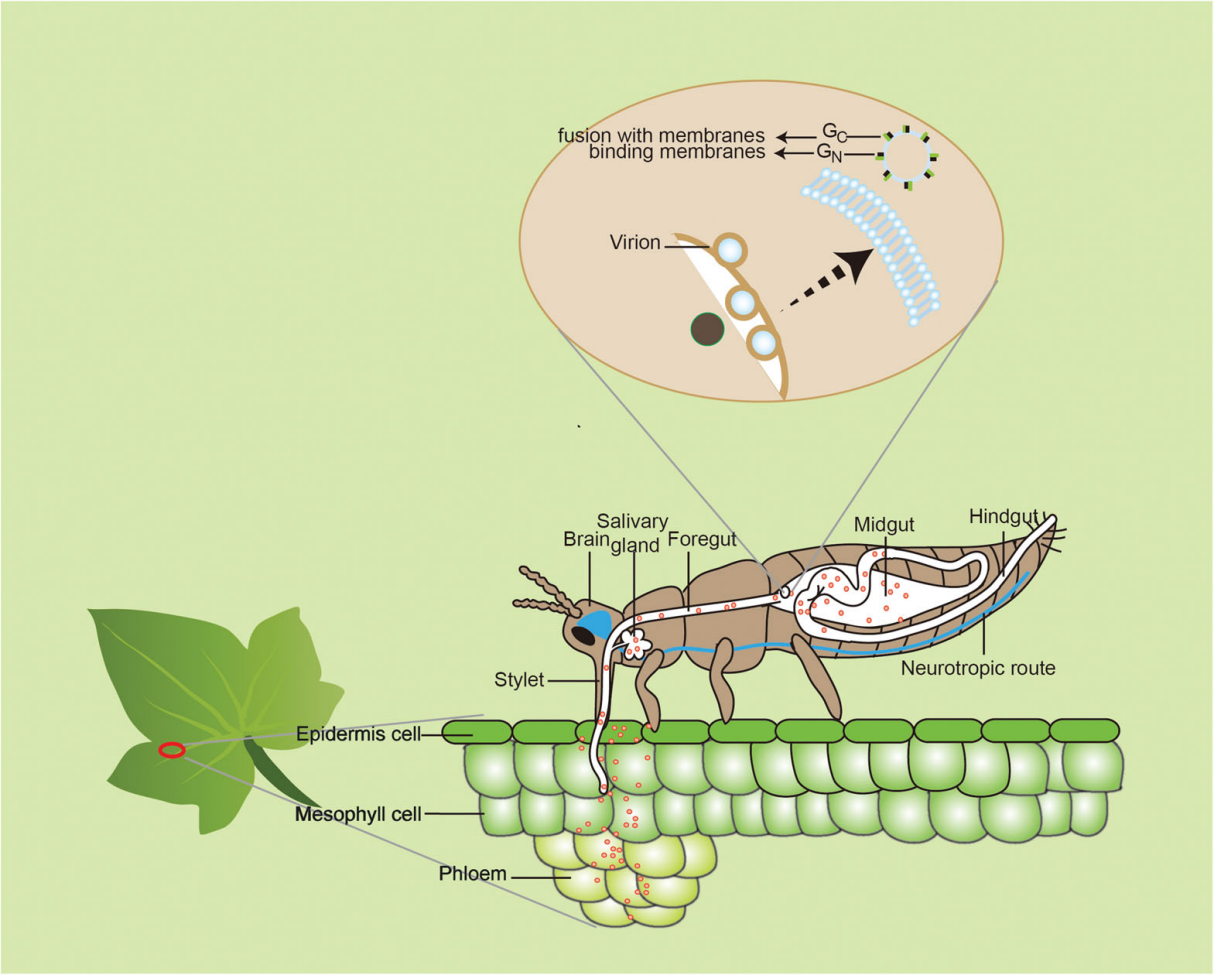
Virus localization sites in *Frankliniella occidentalis*. Viruses are initially acquired through the stylets. They then travel across the alimentary canal to the anterior region of the midgut (MG), where the surface glycoproteins, Gn and Gc, facilitate their entrance into the thrips’ MG. Subsequently, the viruses replicate, accumulate in the visceral muscles of the gut, and then spread to the salivary glands through the connective ligaments, where they are then transmitted back to the plants through the stylets.

Thrips are the only known vectors of orthotospoviruses, but only 0.16% of the known species have been implicated in their transmission (Mound, 2004). Thrips transmit viruses belonging to at least four virus groups, including ilarviruses, machlomoviruses, alphacarmoviruses and orthotospoviruses (Fig. 2, Table S2). (Jones, 2005; Morse & Hoddle, 2006). Using TSWV as an example, this particular virus, which is one of the most economically important members of the genus *Orthotospovirus* (*Tospoviridae*) (Mumford *et al*., 1996), has long been associated with *F. occidentalis* – one of the most important and efficient vector thrips (Wijkamp, 1995a; Arthurs *et al*., 2018a). TSWV acquisition by *F. occidentalis* is a developmental‐stage dependent process, with the 1st instar larval stage considered as the most susceptible phase (Rotenberg *et al*., 2015). The interactions between TSWV and *F. occidentalis* and its dissemination route in thrips has been thoroughly reviewed by Whitfield *et al*. (2015a), Rotenberg *et al*. (2015) and Dietzgen *et al*. (2016). TSWV is acquired by the thrips’ stylets and travels across the alimentary canal to the anterior region of the midgut (MG), where the surface glycoproteins, Gn and Gc, facilitate its entrance into the thrips’ MG (Whitfield *et al*., 2004, 2005b). Subsequently, TSWV replicates and accumulates in the visceral muscles of the gut, later spreading back into the salivary glands through the connected ligaments, and is then transmitted to the plant by the stylets (Fig. 3). Abe *et al*. (2011) suggested that TSWV infection facilitates the production of thrips of the next generation, which will contribute to further spread of TSWV. Stafford *et al*. (2011) reported that TSWV infection directly influences the feeding behavior of thrips, and enhances the transmission efficiency of the virus, whereas, viruses such as ilarviruses are also thought to be transmitted very transiently by *F. occidentalis* (Aramburu *et al*., 2010), where the transmission starts when the thrips feed on virus‐laden pollen and ends once the virus‐laden pollen is gone.

## Current global status of the viruses transmitted by *F. occidentalis*

Presently, a total of 11 viruses have been reported vectored by *F. occidentalis*. These include eight species in the genus *Orthotospovirus* (*Tospoviridae*): alstroemeria necrotic streak orthotospovirus (AlNSV), chrysanthemum stem necrosis orthotospovirus (CSNV), groundnut ringspot orthotospovirus (GRSV), impatiens necrotic spot orthotospovirus (INSV), tomato chlorotic spot orthotospovirus (TCSV), TSWV, tomato yellow ring virus (TYRV), and tomato zonate spot orthotospovirus (TZSV); parietaria mottle virus (PMoV) in the genus *Ilarvirus* (*Bromoviridae*); and pelargonium flower break virus (PFBV) in the genus *Alphacarmovirus*, and maize chlorotic mottle virus (MCMV) in the genus *Machlomovirus* (both in *Tombusviridae*) (Fig. 2, Table S2). The global distributions, hosts, emergence and dissemination of these viruses are discussed below.

### AlNSV

AlNSV was first described in Colombia, when it was found to cause necrotic streaks on the leaves of Peruvian lilies (*Alstroemeria* sp.) (Hassani‐Mehraban *et al*., 2010). According to the nucleocapsid (N) protein gene sequence, phylogenetic analysis revealed that AlNSV clustered with those orthotospoviruses from the American continent into a single lineage with a significantly close serological relationship (Hassani‐Mehraban *et al*., 2010; Liu *et al*., 2017). Similar to other reference orthotospoviruses, AlNSV is capable of infecting ornamentals as well as vegetables locally or systemically, and is transmitted by *F. occidentalis* under experimental conditions (Hassani‐Mehraban *et al*., 2010).

### CSNV

CSNV was first identified from chrysanthemums in Brazil in 1996 (Resende *et al*., 1996), followed by the Netherlands, Slovenia, UK, Japan and South Korea (Resende *et al*., 1996; Verhoeven *et al*., 1996; Mumford *et al*., 2003; Ravnikar *et al*., 2003; Okuda *et al*., 2013; Yoon *et al*., 2017a). In Brazil, CSNV also infects tomatoes with necrosis and necrotic spots on the stem and leaves, showing symptoms similar to those seen in chrysanthemums (Bezerra *et al*., 1999; Nagata *et al*., 2004). *F. schultzei* and *F. intonsa* have also been identified as vectors of CSNV although their efficiencies as vectors are much lower than *F. occidentalis* (Nagata *et al*., 2004; Okuda *et al*., 2013).

### GRSV

GRSV was first described from South Africa and Brazil from peanuts and tomatoes, respectively (Wijkamp, 1995b; Pappu *et al*., 2009). It was subsequently reported from Argentina, the USA, the Caribbean basin and Ghana from a relatively narrow host range compared to TSWV (Webster *et al*., 2010; Webster *et al*., 2011; Camelo‐Garca *et al*., 2014; Spadotti *et al*., 2014; Leão *et al*., 2015; Webster *et al*., 2015; Appiah *et al*., 2016). In Brazil, GRSV was identified from several hosts including sweet peppers, coriander, cocona, cucumbers, cubiu, peanuts and watermelons (Lima *et al*., 1999; Boari *et al*., 2002; Camelo‐Garca *et al*., 2014; Spadotti *et al*., 2014; Leão *et al*., 2015). In North America, GRSV was initially reported from tomatoes in south Florida in 2009, subsequently from peppers, tomatilloes and eggplants in peninsular Florida, and later in South Carolina and New York (Webster *et al*., 2010, 2011, 2015). Interestingly, a reassortant isolate GRSV‐L_G_M_T_S_G_, composed of the L and S RNAs from GRSV and the M RNA from TCSV, was reported from tomatoes in Florida in 2010 (Webster *et al*., 2011). The recent outbreaks of GRSV in Brazil and North America were probably driven by its major thrip vectors, *F. occidentalis*, *F. schultzei*, and *F. gemina* (Pappu *et al*., 2009; Gilbertson *et al*., 2015; Webster *et al*., 2015). Among the three species involved, *F. schultzei*, the local species from Brazil and North America, has a more efficient transmission than the other two thrip species (Nagata *et al*., 2004; Gilbertson *et al*., 2015; Webster *et al*., 2015).

### INSV

INSV, which is considered to be an important pathogen of ornamental crops, was initially characterized and distinguished from New Guinea impatiens in the Netherlands in the late 1980s (Ávila *et al*., 1992). It is now widespread throughout much of the world (Vaira *et al*., 1993; Peters *et al*., 1996; Lebas & Ochoa‐Corona, 2007; Pappu *et al*., 2009). In northern Africa, the Middle East, Southeast Asia, southern New Zealand, the Caribbean and Central America, INSV has been reported from numerous field and greenhouse‐grown ornamentals, including freesia, impatiens, lobelia, primula, ranunculus, begonia, chrysanthemum, and so on, (Lebas *et al*., 2004; Jones, 2005; Lebas & Ochoa‐Corona, 2007; Pappu *et al*., 2009), as well as a number of weed species (Okuda *et al*., 2010). Traditionally, INSV was also believed to be a pathogen on some vegetable crops, although it is only capable of causing limited local symptoms or is symptomless on sweet peppers, pepinos, spinach, tomatoes and cucumbers (Verhoeven & Roenhorst, 1998; Vicchi *et al*., 1999; Sialer & Gallitelli, 2000; Mavric & Ravnikar, 2001). However, INSV has recently emerged as an important pathogen of lettuce caused by *F. occidentalis* transferring from ornamental hosts in coastal regions of California (Pappu *et al*., 2009; Kuo *et al*., 2014; Gilbertson *et al*., 2015). In addition to *F. occidentalis*, INSV can also be transmitted by *F. intonsa* and *F. fusca*, but with a lower efficiency (Naidu *et al*., 2001; Sakurai *et al*., 2004).

### TCSV

In 1990, TCSV was first characterized as a distinct serotype of TSWV from tomatoes in Brazil (De Avila *et al*., 1990, 1993). Subsequently, TCSV was isolated from sweet peppers, potatoes, endives, celery, lisianthus and various weeds with mosaic, necrosis, chlorotic or stunting symptoms in Argentina and Brazil (Boiteux *et al*., 1993; Gracia *et al*., 1999; Colariccio *et al*., 2001a; Dal Bio *et al*., 2001; Eiras *et al*., 2002), and from outbreaks on lettuce and gilo in Brazil (Colariccio *et al*., 2001b; Rabelo *et al*., 2002). In the USA, TCSV was first detected from tomatoes in south Florida in 2012 (Londoño *et al*., 2012), and then from lettuce, impatiens and peppers (Webster *et al*., 2015). In Puerto Rico, TCSV was also found from tomatoes, peppers, jimsonweed (*Datura stramonium*), and lettuce in 2013 (Estévez de Jensen *et al*., 2013; Estévez de Jensen & Adkins, 2014). More recently, TCSV was identified from tomatoes in the Dominican Republic (Batuman *et al*., 2014).

### TSWV

At the beginning of the 20th century, spotted wilt disease of tomato was first described in Australia in 1915 (Brittlebank, 1919). Afterwards, it was considered as a viral disease caused by TSWV and transmitted by *T. tabaci* and *F. schultzei* (Pittman, 1927; Samue *et al*., 1930). Normally, TSWV‐infecting tomatoes shows bronzing, curling necrotic streaks and spots on the leaves, and a paler red or yellow skin color on the fruits. In addition to tomatoes, TSWV was subsequently isolated from many other plants such as aubergine, artichoke, bell peppers, cabbage, chrysanthemums, cowpeas, cucumbers, butternut squash, hot peppers, common beans, lettuce, petunias, papaya, peas, peanut, eggplants, pineapples, potatoes, strawberries, mangos, soybeans, celery, spinach, sweet peppers, broad beans, tobacco, cauliflower and assorted weeds (Chatzivassiliou *et al*., 2000a; Jones, 2005; Whitfield *et al*., 2005a; Reitz *et al*., 2011).

In Europe, TSWV was first described in England in 1929 from ornamental winter cherries (*Solanum capsicastrum*) with concentric ring symptoms on leaves (Smith, 1932). In the early 20th century, TSWV was transmitted mainly by *T. tabaci*. An obvious decline of TSWV‐infecting crops was evident when effective controls successfully managed this vector. When *F. occidentalis* extended its range to Europe, TSWV began to be a major threat to European horticultural crops (Jones, 2005). Today, TSWV is well established in almost all European countries, including Albania (Cota & Merkuri, 2004), Bulgaria (Dikova *et al*., 2013), Bosnia and Herzegovina (Trkulja *et al*., 2013), France (Marchoux *et al*., 1991), Greece (Chatzivassilou *et al*., 1996; Chatzivassiliou *et al*., 2000b), Hungary (Salamon *et al*., 2012), Montenegro (Zindović *et al*., 2011), Spain (Jorda, 1993; Aramburu *et al*., 1997), Portugal (Louro, 1996), and Slovenia (Mavric & Ravnikar, 2001). In Serbia, TSWV was isolated from *Gerbera hybrida* in 2009 (Stanković *et al*., 2011), onions, garlic and chrysanthemums in 2011 (Stanković *et al*., 2012, 2013), and *Brugmansia* sp. in 2012 (Nikolić *et al*., 2013).

In Africa, TSWV was first described from a wilt disease of tobacco in South Africa as early as 1905 (Moore, 1933), and later found in several provinces of the country infecting tobacco, tomatoes, peppers and potato crops (Moore & Andessen, 1939). After *F. occidentalis* was introduced into Africa, TSWV became widespread in other African countries (Moussa *et al*., 2000; Ben Moussa *et al*., 2005). Recently, TSWV has been found from *Amaranthus thunbergii* in South Africa (Kisten *et al*., 2016), butternut squash (*Cucurbita moschata*) and peppers in Zimbabwe in 2015 (Karavina *et al*., 2016a,b).

In Asia, TSWV was first recorded in the Middle Eastern countries. In July 1998, TSWV was identified from *Pittosporum tobira* shrubs with foliar ring spots, mild mosaic, and tip necrosis symptoms in a nursery in the Sharon Valley of Israel (Gera *et al*., 2000a), and later from several vegetables (Gera *et al*., 2000b). Similarly, the virus was also detected from potatoes in 1998 in Iran (Pourrahim *et al*., 2001), and subsequently from soybeans, tomatoes, and cucurbits (Golnaraghi *et al*., 2001; Massumi *et al*., 2007, 2009). TSWV has also been isolated from many important vegetable crops in Jordan and Lebanon (Anfoka *et al*., 2006; Abou‐Jawdah *et al*., 2006). More recently, TSWV was reported from lettuce showing necrotic lesions, necrosis of the lamina of the younger leaves, and leaf curling symptoms in March 2014 from the Al‐Uyaynah area, in the central region of Saudi Arabia. In eastern Asia, TSWV is now widely distributed in China, Korea, and Japan in a number of vegetable and horticultural crops such as celery, peppers, cowpeas, lettuce, *Bidens pilosa*, tomatoes, potatoes, *Brugmansia suaveolens*, *Eustoma grandiflorum*, and miscellaneous other wild plant species (Choi *et al*., 2004; Zheng *et al*., 2010; Reitz *et al*., 2011; Okazaki *et al*., 2007, 2011; Choi & Choi, 2015; Li *et al*., 2015; Xiao *et al*., 2016; Yoon *et al*., 2017b). In India, TSWV was found on sunflowers exhibiting severe mosaic, systemic necrosis, leaf distortion, and ringspots symptoms in Tirupati in January 1998 (Subbaiah *et al*., 2000), and more recently from chrysanthemums grown in the Nilgiris district of Tamil Nadu State in August 2013 (Renukadevi *et al*., 2015).

In America and the Caribbean, TSWV was first discovered from pineapples causing yellow spot disease as early as 1926 in Hawaii (Kucharek *et al*., 2000). In the 1970s, the virus was reported from peanuts in Texas (Haliwell & Philley, 1974), and then from peppers, tobacco and tomatoes in Georgia and other areas of the southeastern USA in the mid‐1990s (Culbreath *et al*., 1991). In the latest 20 years, TSWV has become widespread throughout most of the states in the USA (Groves *et al*., 1998; Holcomb *et al*., 1999; Díaz‐Pérez & Pappu, 2000; Holcomb & Valverde, 2000; Momol *et al*., 2000; Adkins *et al*., 2003; Whitfield *et al*., 2003; Mullis *et al*., 2004; Yang *et al*., 2004; Adkins & Baker, 2005; Mullis *et al*., 2006; Nischwitz *et al*., 2006a,b; Baker *et al*., 2007; Baker *et al*., 2009; Barkley *et al*., 2009; Crosslin *et al*., 2009) causing significant economic losses (Pearce, 2005). More recently, TSWV was isolated from *Stevia rebaudiana* and tomatoes with the *Sw‐5* orthotospovirus‐resistance gene in Carolina (Koehler *et al*., 2016; Batuman *et al*., 2017). TSWV was found in a commercial chrysanthemum field of Mexico infesting several weeds including *Taraxacum officinale*, *Bidens* sp., *Reseda luteola*, *Mirabilis jalapa* being transmitted by *F. occidentalis* (Martinez *et al*., 1999). In 2005 to 2006, tomatoes showing chlorosis, malformation of apical leaves, stunting, and ringspot lesions caused by TSWV were first noticed in the Baja California peninsula of Mexico (Holguín‐Peña & Rueda‐Puente, 2007). In the Dominican Republic, TSWV transmitted by *F. occidentalis* was found to be widely distributed in commercial peppers and tomatoes growing under protected greenhouse conditions (Martínez *et al*., 2014). In South America, tomato, pepper, and lettuce crops infected by TSWV were reported from Argentina, Brazil, Chile, and Venezuela causing a significant threat to the vegetable industry (Maluf *et al*., 1991; Gracia *et al*., 1999; EPPO, 2004; Lebas & Ochoa‐Corona, 2007; Rosales *et al*., 2007; Marys *et al*., 2014; Pérez‐Colmenares *et al*., 2015).

The first recognition that tomato spotted wilt disease was caused by TSWV occurred in Australia as early as 1915, although it was considered as an introduction from elsewhere following European colonization (Brittlebank, 1919; Pittman, 1927; Samuel *et al*., 1930; Smith, 1932). In Australia, TSWV was mainly transmitted by *T. tabaci* and *F. shultzei* on several vegetables with limited spread occurring over a span of many decades. When *F. occidentalis* was detected in southwestern Australia in 1993, an outbreak of TSWV was being reported in eastern and southeastern Australia (Latham & Jones, 1997; Wilson *et al*., 2000; Pappu *et al*., 2009). Similarly, TSWV was detected from tomatoes and other vegetables in New Zealand soon after its discovery on the Australian continent, and, at the time, was also transmitted by *T. tabaci* (Chamberlain & Taylor, 1936, 1938). More recently, a serious epidemic of TSWV on ornamental plants grown in greenhouses on the North Island was caused by *F. occidentalis* rather than *T. tabaci* (Fletcher *et al*., 2005; Pappu *et al*., 2009).

### TYRV

TYRV, a tentative orthotospovirus species, is closely related to iris yellow spot virus. The virus was first identified from tomatoes in Teheran Province, Iran (Hassani‐Mehraban *et al*., 2005). Subsequently, TYRV was isolated from chrysanthemums, gazanias, potatoes, soybeans, and cineraria with high diversity in the N gene in Iran (Hassani‐Mehraban *et al*., 2005, 2007; Rasoulpour & Izadpanah, 2007). In 2012, TYRV was isolated from tomatoes with chlorotic ring spots on fruits and necrosis of stems and leaves in Kenya (Birithia *et al*., 2012). TYRV has now been found in Europe in tomato plants having symptoms of necrosis on leaves and stalks, and chlorotic and necrotic ringspots on fruits in Kujawsko‐Pomorskie Province, Poland (Zarzyńska‐Nowak *et al*., 2016).

### TZSV

TZSV was first reported to naturally infect tomatoes, causing zoned ring spots on fruits in Yunnan Province, China (Dong *et al*., 2008). In Yunnan Province, TZSV was subsequently isolated from chili peppers (*Capsicum annuum*), peppers, tobacco, *Iris tectorum*, potatoes and several weeds, including *Bidens pilosa* and *Rumex dentatus* (Dong *et al*., 2010; Zheng *et al*., 2014; Huang *et al*., 2015; Liu *et al*., 2015; Wu *et al*., 2016a). During a survey from 2008 to 2010 in Guangxi Province, China, TZSV was also detected from tobacco. Infection symptoms included dwarfing, midrib browning, distorted apical buds, and concentric ringspot (Cai *et al*., 2011).

### PMoV

PMoV, a member of the genus *Ilarvirus*, was originally isolated from the weed *Parietaria officinalis* in 1989 (Caciagli *et al*., 1989), and afterwards from tomato, *Mirabilis jalapa*, *Capsicum annuum*, *Diplotaxis tenuifolia* in Italy (Roggero *et al*., 2000; Parrella, 2002; Parrella *et al*., 2016, 2017). Besides Italy, PMoV has also been detected from tomatoes in France, Spain and Greece, and from *Capsicum annuum* in Spain (Ramasso *et al*., 1997; Roggero *et al*., 2000; Aramburu, 2001; Galipienso *et al*., 2005; Janssen *et al*., 2005).

### PFBV

PFBV is a member of the genus *Alphacarmovirus*, affecting *Pelargonium* spp. which causes white flower streaking, chlorotic spotting of leaves and stunting on some cultivars. It was originally identified in Europe and has now spread throughout much of the world (Stone & Hollings, 1973; Bouwen & Maat, 1992; Blystad *et al*., 1995; Ivars *et al*., 2004; Rico *et al*., 2004; Rico & Hernández, 2006; Rico *et al*., 2006; Wei *et al*., 2015). It is primarily known for its detrimental effects on the production and quality of some *Pelargonium* spp. cultivars (Bouwen & Maat, 1992; Blystad *et al*., 1995; Krczal *et al*., 1995; Ivars *et al*., 2004; Wei *et al*., 2015). PFBV is frequently transmitted and dispersed by vegetative propagation and irrigation systems as well as by the western flower thrip, *F. occidentalis* (Krczal *et al*., 1995).

### MCMV

MCMV, a member the genus *Machlomovirus* in the family *Tombusviridae*, was first identified from maize in the Americas including plants from Peru and the USA (Castillo‐Loayza, 1977; Niblett & Clafin, 1978; Jiang *et al*., 1990). In maize, MCMV is among the important pathogens that characteristically induce typical symptoms in the plants such as mosaicism, stunting and necrosis (Niblett & Clafin, 1978; Mahuku *et al*., 2015; Chen *et al*., 2017). MCMV, together with other maize‐infecting potyviruses, are responsible for inducing corn lethal necrosis disease, which was first described in Peru in 1974 and has since spread worldwide (Castillo‐Loayza, 1977; Niblett & Clafin, 1978; Morales *et al*., 1999; Adams *et al*., 2014; Deng *et al*., 2014; Lukanda *et al*., 2014; Gowda *et al*., 2015; Mahuku *et al*., 2015; Quito‐Avila *et al*., 2016; Chen *et al*., 2017). In addition to maize, MCMV also can infect sorghum, Coix seed and finger millet in several Asia and Africa regions, probably due to its diverse transmission methods including by seeds, mechanical inoculation, and insects including thrips and beetles (Jiang *et al*., 1990; Cabanas *et al*., 2013; Deng *et al*., 2014; Kusia *et al*., 2015; Achon *et al*., 2017; Chen *et al*., 2017).

In conclusion, from the emergence and dissemination of *F. occidentalis* and its transmitting viruses, we speculate that the western flower thrip has spread from its original distribution in western North America to tropical, subtropical, temperate and cold temperate zones of the world by the movement of horticultural material, such as cuttings, seedlings and potted plants, while the spread of *F. occidentalis‐*transmitting viruses is shared along with the migration pattern (or trends) of its vector, especially TSWV and INSV. *F. occidentalis* was rarely reported in cold zones especially in areas with temperature dropping to −10 °C in winter which is 100% lethal to *F. occidentalis* attempting to overwinter outdoors.

## Management of *F. occidentalis*

Because of their small size and the difficulty involved in detection and identification, successful invasions of thrips often occur unnoticed. As a result, *F. occidentalis* has become a major global pest with immense damage potential in only 30 years. In addition, adults are capable of migrating long distances to new host plants and are able to quickly transmit their viruses (Kliot *et al*., 2016). With these risks in mind, the most effective means of dealing with this potentially invasive and pestiferous thrip species is to prevent its entry and establishment into nonendemic regions. For example, methyl bromide was used for post‐harvest fumigation of a number of commodities either by the exporting or importing country (provinces) after a thrip infestation is noticed (Morse & Hoddle, 2006). However, because of its ozone depletion effect, methyl bromide is being phased out worldwide (Deewatthanawong *et al*., 2016). The alternatives to methyl bromide include irradiation, sulfuryl fluoride, phosphine, ethanedinitrile, low oxygen treatments, heat and cold treatments (Cox, 2017). A number of sustainable tactics have been developed in IPM programs for managing *F. occidentalis* and to inhibit its persistent spread worldwide with ever increasing damage to its many host crops.

### Chemical control

Management of *F. occidentalis* has been a difficult task. Use of insecticides has traditionally been the primary strategy for control of *F. occidentalis*, especially in virus‐sensitive crops (Bielza, 2008). The insecticides that are normally applied can be separated into two major groups: broad‐spectrum insecticides, which include pyrethroids, neonicitinoids, organophosphates and carbamates, and narrow‐spectrum insecticides, which include pyridalyl and lufenuron (Mouden *et al*., 2017). However, frequent applications of insecticides, especially those containing pyrethroids, organophosphates, neonicitinoids and carbamates have also decimated large percentages of natural enemies and led to the rapid development of insecticide resistance in *F. occidentalis* (Mouden *et al*., 2017). This propensity of *F. occidentalis* for developing insecticide resistance has been a primary factor in promoting its pest status.

Spinosad and the related spinetoram, which tend to be compatible with natural enemies, are now being extensively used and currently provide the most effective chemical control of *F. occidentalis* (Gao *et al*., 2012a; Li *et al*., 2016). However, the applications of any insecticide will eventually contribute to resistance development in a given pest species. Evidence has shown that Spinosad resistance is now present in some populations of *F. occidentalis* in the USA (Weiss *et al*., 2009), Australia (Herron *et al*., 2014) and China (Li *et al*., 2016). If deemed necessary, insecticide use should be accurate, precise and complement other compatible control approaches.

### Agricultural practices

Thrips often overwinter in patches of uncultivated plants and migrate into cropping systems in the spring (Pearsall & Myers, 2000), with cropping systems often serving as a sink, with sources of insect populations occurring in field margins and fencerows. *F. occidentalis*, which is a highly polyphagous pest of many cultivated as well as wild plants, has been shown to feed on more than 240 host plants (Tommasini & Maini, 1995), including many weed species. In France, the weed, gallant soldier (*Galinsoga parviflora* Cav.), has been reported by Nyasani *et al*. (2013) as an excellent host of *F. occidentalis* for both feeding and reproduction under field conditions, and serves as a potential source of thrip outbreaks in French bean fields. In instances such as this, where alternative hosts are identified and known to be present, sound agricultural practices such as seasonal mowing of these weeds will likely decrease the number of thrips migrating into the cropping systems (Northfield *et al*., 2008).

Additional practices for managing *F. occidentalis* include creating a less favorable environment by irrigation to reduce numbers of *F. occidentalis* adults (Schuch *et al*., 1998), by decreasing the levels of nitrogen fertilization to reduce populations of *F. occidentalis* in ornamentals (Brodbeck *et al*., 2001; Chow *et al*., 2012), and by growing trap plants to draw *F. occidentalis* away from susceptible crops, thereby reducing the number of thrips on the target crop (Cook *et al*., 2006).

### Physical control

Because of their small size, fine mesh screens have been widely used to cover greenhouse openings such as vents to help physically prevent thrips from immigrating onto protected crops (Arthurs *et al*., 2018b). It was reported by Tinoco *et al*. (2014) that using appropriate mesh size screens on greenhouse windows would reduce the incidence of *F. occidentalis* by 20% in protected tomatoes. Because thrips find suitable host plants by utilizing different cues, including visual cues in the ultraviolet (UV) spectrum (Terry, 1997), using materials that reflect UV radiation can obscure their host‐locating cues. Several researchers have found that using UV‐reflective mulch significantly reduced early season abundance of adult thrips and disease incidence (Stavisky *et al*., 2002; Kigathi & Poehling, 2012).

Another conventional measure, sticky cards, are widely used by growers for monitoring thrip populations in greenhouses (Ren *et al*., 2008). It was reported that blue cards are highly attractive to *F. occidentalis* (Otieno *et al*., 2018). Because adult thrips explore their host range in part through volatiles, the commercially available *F. occidentalis* semiochemicals are frequently used as lures in conjunction with sticky card traps (Broughton *et al*., 2015) to attract and monitor or eliminate thrips.

### Biological control

There has been considerable interest in the use of biological control agents to reduce thrip populations, especially in protected crops. An effective use of agents has been shown to improve thrip management. Inoculative release of agents, beginning at crop initiation before the resident thrips approach economically damaging levels, is recommended (Reitz *et al*., 2011). The large number of biological control agents that have been reported to attack *F. occidentalis* can be separated into two groups: macrobials (predators and parasitoids) and microbials (fungal pathogens and entomopathogenic nematodes) (Mouden *et al*., 2017). The macrobials currently being widely and effectively used are anthocorid bugs (*Orius* spp.) (Mo *et al*., 2013; Aragón‐Sánchez *et al*., 2018), green lacewing species (Sarkar *et al*., 2019) and predatory phytoseiid mites (Messelink *et al*., 2006; Ahmed & Lou, 2018), which predominantly attack 1st instar thrips on foliage, and soil‐dwelling predaceous laelapid mites (Berndt *et al*., 2004; Wu *et al*., 2016b), which consume thrip pupae in soil.

Fungal pathogens used as biocontrol agents of *F. occidentalis*, are *Beauveria bassiana* (Gao *et al*., 2012b; Lee *et al*., 2017), *Metarhizium anisopliae* (Maniania *et al*., 2003; Toledo‐Hernández *et al*., 2017) and *Lecanicillium lecanii* (Gouli *et al*., 2009; Wang *et al*., 2013). The various nematode species used against soil‐inhabiting pupae of *F. occidentalis* are in the genera *Steinernema* and *Heterorhabditis* (Ebssa *et al*., 2004; Buitenhuis & Shipp, 2005). There is currently interest in combinations of different biological control agents, which may result in an additive suppression of *F. occidentalis* populations (Messelink & Janssen, 2014; Saito & Brownbridge, 2016; Wu *et al*., 2017). Furthermore, fungal‐based granular formulations of entomopathogenic fungi are regarded as an effective strategy for thrip management by controlling the soil‐dwelling developmental stages of thrips (Lee *et al*., 2017). The latest research demonstrates that the application of *B. bassiana* granules to the soil surface can successfully suppress *F. occidentalis* under greenhouse conditions (Zhang *et al*., 2019).

## Concluding remarks

With the continuing increase in global trade in ornamental greenhouse plants, it is likely that *F. occidentalis* will continue its rapid spread into, as yet, uninfested areas around the world, causing substantial amounts of damage from feeding and virus transmission. Another consideration is that *F. occidentalis* may also be capable of expanding its range to new areas as a consequence of global climate warming (Wu *et al*., 2018). Considering the economic importance of *F. occidentalis* both as a pest and a vector of several notorious plant viruses, it is essential to establish early surveillance systems of the species and to encourage the rapid recognition of plant virus symptoms while keeping a constant vigil on further spread of the species, especially in cold zones where *F. occidentalis* has not been reported.

Aggregation pheromones of *F. occidentalis* have been identified and shown to be cost‐effective for monitoring detection of this thrip species in the field (Kirk, 2017). Huang *et al*. (2010) and Zhang *et al*. (2012) provided a diagnostic polymerase chain reaction detection system, which can quickly and accurately identify *F. occidentalis* from thrip larvae to complement the traditional morphological identification. This method can also be used for on‐site testing of samples at ports‐of‐entry in the future. Standard area diagrams (SADs) have been used as a tool to improve the accuracy and reliability of visual estimates of leaf spotting diseases. More than 100 diseases with a range of plant organs were validated by SAD (Del Ponte *et al*., 2017). Presently, enhanced accessibility of cameras and image analysis software has accelerated the development of more realistic, stylized color representations or diagrams based on photographs of diseased plant organs (Del Ponte *et al*., 2019). Hence, we consider that it is a potential method for rapid recognition of *F. occidentalis*‐transmitting virus symptoms in the near future.

Most of the vector thrip species have high fecundity, short reproductive cycles and extensive plant host ranges (Whitfield *et al*., 2005a). *F. occidentalis* populations tend to be efficient vectors of multiple orthotospovirus species. Although the research on biological processes involved in the transmission of orthotospoviruses and their thrip vectors has made progress during the last decade (Hogenhout *et al*., 2008; Rotenberg *et al*., 2015), there is still a lack of effective measures for management of *F. occidentalis* and its transmitted viruses. At present, there is still a heavy reliance on insecticides, which will continue to play an important role in thrip management in the foreseeable future. The increased incidence of *F. occidentalis* throughout the world that we are currently witnessing, could be a consequence of increased insecticide applications over the past 30 years. There is mounting evidence that synthetic pyrethroids can stimulate reproduction of *F. occidentalis* (Funderburk, 2009) and promote insecticide resistance (Gao *et al*., 2012a). Further studies, including the virus‐vector relationship of *F. occidentalis* with insecticide resistance are needed to improve our understanding of basic biological concepts and develop alternative measures for thrip control.

Although significant research progress in control of *F. occidentalis* has been made after using alternative measures, this thrip species continues to threaten the production of many crops worldwide because of the severity of viruses and difficulty in preventing thrip transmission. Therefore, both the thrips and plant virus diseases transmitted should be taken into account in control tactics. First, because immature *F. occidentalis* can be found consistently in tomato blossoms (Beaudoin, 2011) and have been shown to acquire TSWV from infected tomato and then transmit to susceptible plants (Szostek *et al*., 2017), it was suggested that management of *F. occidentalis* infestations during the blooming season may be important for effective control of TSWV in susceptible tomato cultivars (Houle & Kennedy, 2017). Second, because adult thrips oviposit in plant tissue and prefer tight spaces, contact insecticides are often not effective against thrips. Induced systemic resistance (ISR) has recently gained more interest and might be the important option for management of thrips and transmitted virus (Mouden *et al*., 2017). It was reported that *Pseudomonas* strains induced resistance against virus diseases (Vasanthi *et al*., 2010); the combination of ISR by *Pseudomonas* and Neem oil, would be a best alternative in the future (Vasanthi *et al*., 2017). In addition, integration of new biotechnology‐based strategies, as well as advances in computational systems will provide a powerful tool to drive innovation in reducing virus transmission and vector populations.

## Disclosure

The authors declare no competing interests.

## Supporting information


**Table S1**. The worldwide distribution of *Frankliniella occidentalis*.

**Table S2**. The worldwide distribution and host of viruses transmitted by *Frankliniella occidentalis*.
