# Supplementary material for: A global invasion by the thrip, Frankliniella occidentalis: Current virus vector status and its management
Source: Insect Sci. 2019 Oct 23;27(4):626–45. doi: 10.1111/1744-7917.12721 (PMC7318653; doi:10.1111/1744-7917.12721)
Supplement: Supplementary file 1 — Table S1. The worldwide distribution of Frankliniella occidentalis. [file INS-27-626-s001.docx]

Table S1. The worldwide distribution of *Frankliniella* *occidentalis*

| **Country** | **References** |
| --- | --- |
| Albania | Çota and Merkuri 2004 |
| Algeria | Benmessaoud-Boukhalfa *et al*. 2010 |
| Argentina | de Breuil *et al*. 2015 |
| Argentina | Carrizo 1998 |
| Australian | Stobbs *et al*. 1992 |
| Belgium | Verhoyen and Gofflot 1990 |
| Britain | Mcdonald *et al*. 1998 |
| Bulgaria | Hristova *et al*. 2001 |
| Bulgaria | Krumov and Karadjova 2012 |
| Canada | Pearsall 2002. |
| Chile | Ripa *et al*. 2009 |
| China | Zhang *et al*. 2003 |
| Colombia | Ripa *et al*. 2009; Ebratt *et al*. 2013 |
| Costa Rica | Wang *et al*. 2010 |
| Czech | Mertelik *et al*. 1996 |
| Dominican Republic | Wang *et al*. 2010 |
| Ecuador | Ripa *et al*. 2009 |
| Finland | Rautapää 1992 |
| France | Marchoux *et al*. 1991 |
| Georgia | Chamberlin *et al*. 1992 |
| Germany | Ascher *et al*. 1992 |
| Greece | Chatzivassiliou *et al*. 2000 |
| Guatemala | Wang *et al*. 2010 |
| Hungary | Jense *et al*. 2009 |
| Indonesia | Wang *et al*. 2010 |
| Iran | Mirabbalou 2013 |
| Ireland | Dunne and O'Connor 1989 |
| Israel | Argaman *et al*. 1989 |
| Japan | Katayama *et al*. 2000 |
| Kenya | Macharia 2015 |
| Malaysia | Wang *et al*. 2010 |
| Martinique | Wang *et al*. 2010 |
| Mexico | Wang *et al*. 2010 |
| Morocco | Bounfour *et al*. 2005 |
| Netherlands | Dissevelt *et al*. 1995; Mirnezhad *et al*. 2012; Pijnakker and Ramakers 2008 |
| New Zealand | Martin and Workman1994 |
| Peru | Ripa *et al*. 2009 |
| Philippines | Wang *et al*. 2010 |
| Poland | Labanowski 1992 |
| Portugal | Frescata and Mexia 1995 |
| Puerto Rico | Wang *et al*. 2010 |
| Qatar | Mirab-balou *et al*. 2014 |
| Romania | Bărbuceanu and Vasiliu-Oromulu 2012 |
| Singapore | Wang *et al*. 2010 |
| SouthAfrica | Wang *et al*. 2010 |
| South Korea | Han *et al*. 1998 |
| Spain | Espinosa *et al*. 2002 |
| Sri Lanka | Tillekaratne *et al*. 2010 |
| Thailand | Wang *et al*. 2010 |
| The Republic of Belarus | Dolmatov *et al*. 2012 |
| Tunisia | Elimem and Chermiti 2009;Elimem *et al*. 2014 |
| United States | Reitz *et al*. 2011 |
| Venezuela | Ripa *et al*. 2009 |
| Vietnam | Wang *et al*. 2010 |
| Zambia | Kapooria and Ndunguru 2004 |

**References**

Argaman, Q., Klein, Z., Ben-Dov, Y. & Mendel, Z.. *Frankliniella occidentalis* (Thysanoptera: Thripidae), an injurious intmderl. Hassadeh **69**, 1268-1269 (1989).

Ascher, K.R.S., Klein, M. & Meisner, J. Azatin, a neem formulation, acts on nymphs of the western flower thrips. *Phytoparasitica* **20**, 305-306 (1992).

Bărbuceanu, D. &Vasiliu-Oromulu, L. Thrips species (Insecta: Thysanoptera) of ornamental plants from the parks and greenhouses of ADP Pitesti//The BIOATLAS Congress. 24-26 May 2012, Transylvania University of Brasov. 33-7 (2012).

Benmessaoud-Boukhalfa, H., Mouhouche, F. & Belmazouzi, F. Z. Inventory and identification of some Thrips species in coastal and subcoastal regions of Algeria. *Agriculture and Biology J. North America* **1**, 755-761 (2010)..

Bounfour, M., Jebbour, F. & Wadjinny, J. Biological traits of invasive insect species harmful to Moroccan agriculture[C]//Proceedings of BCPC Conference (9-11 June 2005). Humbolt University, Germany. 95-100 (2005).

Carrizo, P. I. Wild hosts for vector trips of tomato spotted wilt virus: proposal of risk ranking. Boletin de Sanidad Vegetal. Plagas (Espana) (1998).

Chamberlin, J. R., Todd, J. W., Beshear, R. J., Culbreath, A. K. & Demski, J. W. Overwintering hosts and wingform of thrips, Frankliniella spp., in Georgia (Thysanoptera: Thripidae): implications for management of spotted wilt disease. *Environ. Entomol.* **21**, 121-128 (1992).

Chatzivassiliou, E. K., Livieratos, I., Jenser, G. & Katis, N. I. Ornamental plants and thrips populations associated with tomato spotted wilt virus in Greece. *Phytoparasitica* **28**, 257-264 (2000)..

Çota, E. & Merkuri, J. Introduction of *Frankliniella occidentalis* and occurrence of Tomato spotted wilt tospovirus in Albania. *EPPO Bull. 34*, 421-422 (2004).

de Breuil, S., La Rossa, F. R., Giudici, A., Wulff, A., Bejerman, N., Giolitti, F. & Lenardon, S. Phylogenetic analysis of Groundnut ringspot virus isolates from peanut and identification of potential thrips vectors in peanut crop in Argentina. *Agriscientia* **32**, 77-82 (2015).

Dissevelt, M., Altena, K. & Ravensberg W. J. Comparison of different Orius species for control of *Frankliniella occidentalis* in glasshouse vegetable crops in the Netherlands, **60**, 839-845 (1995).

Dolmatov, D.A., Prishchepa, I.A. & Kazakevich, N.V. Efficiency and peculiarities of insecto-acaricide voliam targo application against protected ground vegetale crop pests. *Agri. Plant Protect.: Scientific-practical J*. (2012).

Dunne, R. & O'Connor, J. P. Some insects (Thysanoptera: Diptera) of economic importance, new to Ireland. *The Irish Nat. J.* **23**, 63-65 (1989).

Ebratt, R., Everth, E., Acosta, A., Martínez, B., Olga, Y., Guerrero, G. & Turizo, A. Tomato spotted wilt virus (TS WV), weeds and thrip vectors in the tomato (Solanum lycopersicum L.) in the Andean region of Cundinamarca (Colombia). *Agro. Colomb*. **31**, 58-67 (2013).

Elimem, M, dA SilvA, J. A. T. & Chermiti, B. Double-attraction Method to Control *Frankliniella occidentalis* (Pergande) in Pepper Crops in Tunisia. *Plant Protect. Sci.* **50**, 90-96 (2014)..

Elimem, M. & Chermiti, B. Population dynamics of *Frankliniella occidentalis* Pergande (1895)(Thysanoptera: Thripidae) and evaluation of its different ecotypes and their evolution in a rose (Rosa hybrida) greenhouse in the Sahline Region, Tunisia. Tunisian Plant Science and Biotechnology I. *Afri. J. Plant Sci. Biotech.* **3**, 53-62 (2009).

Espinosa, P. J., Bielza, P., Contreras, J. & Lacasa, A. Insecticide resistance in field populations of *Frankliniella occidentalis* (Pergande) in Murcia (south‐east Spain). *Pest Manag. Sci.* **58**, 967-971 (2002)..

Frescata, C. & Mexia, A. Biological control of western flower thrips with Orius laevigatus (Heteroptera: Anthocoridae) in organic strawberries in Portugal. In Thrips biology and management (pp. 249-249). Springer, Boston, MA (1995).

Han, M. J. *et al*. Distribution and host plants of recently introduced western flower thrips, *Frankliniella occidentalis* (Pergande)(Thysanoptera: Thripidae) in Korea. *RDA J. Crop Protect.* (1998).

Hristova, D., Karadjova, O., Yankulova, M., Heinze, C. & Adam, G. A survey of tospoviruses in Bulgaria. *J. phytopathol.* **149**, 745-749 (2001).

Jenser, G., Kiss, B. & Takács, A. *Ambrosia artemisiifolia* is a joint host of tomato spotted wilt virus (TSWV) and its vectors, *Thrips tabci* Lindeman and *Frankliniella occidentalis* (Pergande) in Hungary. Növényvédelem **45**, 435-437 (2009).

Kapooria, R. G. & Ndunguru, J. Occurrence of viruses in irrigated wheat in Zambia. *EPPO Bull*. **34**, 413-419 (2004).

Katayama, H., Kobayashi, H. & Katou, K.. Occurrence of western flower thrips *Frankliniella occidentalis* and its accumulation of tomato spotted wilt virus (TSWV) on chrysanthemums and weeds in Shizuoka prefecture//*P. Kansai Plant Protect. Soc.* **42**, 9-14 (2000).

Krumov, V. & Karadjova, O. Influence of climate change on the potential for establishment of *Frankliniella occidentalis* (Thysanoptera: Thripidae) in Bulgaria. *Acta Phytopathologica et Entomologica Hungarica* **47**, 113-116 (2012).

Labanowski, G. S. *Frankliniella occidentalis* on ornamental crops in Poland and its control. *EPPO Bull*. **22**, 367-376 (1992).

Macharia, I. *et al*. Diversity of Thrips species and vectors of Tomato Spotted Wilt Virus in tomato production systems in Kenya. *J. Econ. Entomol.* **108**, 20-28 (2015).

Marchoux, G., Gebre‐Selassie, K. & Villevieille, M.. Detection of tomato spotted wilt virus and transmission by *Frankliniella occidentalis* in France. *Plant Pathol.* **40**, 347-351 (1991).

Martin, N. A. & Workman, P. J. Confirmation of a pesticide-resistant strain of western flower thrips in New Zealand. In Proceedings of the Forty Seventh New Zealand Plant Protection Conference, Waitangi Hotel, New Zealand, 9-11 August, 1994. (pp. 144-148). New Zealand Plant Prot. Soc. (1994).

Mcdonald J R, Bale, J. S. & Walters, K. F. Effect of temperature on development of the western flower thrips, *Frankliniella occidentalis* (Thysanoptera: Thripidae). *Eur. J. Entomol.* **95**, 301-306 (1998).

Mertelik, J., Götzová, B. & Mokrá, V. Epidemiological aspects of tomato spotted wilt virus infection in the Czech Republic. In IX International Symposium on Virus Diseases of Ornamental Plants 432 (pp. 368-375) (1996).

Mirab-balou, M, Yang, S.L. & Tong, X.L. (2014). First record of nine species of thrips (Insecta: Thysanoptera) in Qatar. *Arab J. Plant Protect.* **32**, 278-282.

Mirabbalou, M. A checklist of Iranian thrips (Insecta: Thysanoptera). *Far Eastern Entomologist* **267**, 1-27 (2013).

Mirnezhad, M. *et al*. Variation in genetics and performance of Dutch western flower thrips populations. *J. Econ. Entomol.* **105**, 1816-1824 (2012).

Pearsall, I. A. Daily flight activity of the western flower thrips (Thysan., Thripidae) in nectarine orchards in British Columbia, Canada. *J. Appl. Entomol.* **126**, 293-302 (2002).

Pijnakker, J. & Ramakers, P. Predatory mites for biocontrol of Western Flower Thrips, *Frankliniella occidentalis* (Pergande), in cut roses. *IOBC WPRS Bull*. **32**, 171 (2008).

Rautapää, J. Eradication of *Frankliniella occidentalis* and tomato spotted wilt virus in Finland: a case study on costs and benefits. *EPPO Bull*. **22**, 545-550 (1992)..

Reitz, S. R., Gao, Y. L. & Lei, Z. R.. Thrips: Pests of concern to China and the United States. *J. Integr. Agr.* **10**, 867-892 (2011).

Ripa, R. *et al*. Population abundance of *Frankliniella occidentalis* (Thysanoptera: Thripidae) and natural enemies on plant hosts in central Chile. *Environ. Entomol.* **38**, 333-344 (2009).

Stobbs, L. W., Broadbent, A. B., Allen, W. R. & Stirling, A. L. Transmission of tomato spotted wilt virus by the western flower thrips to weeds and native plants found in southern Ontario. *Plant Dis.* (USA) (1992).

Tillekaratne, K. *et al*. List of thrips (Thysanoptera) recorded from Sri Lanka. *J. National Sci. Foundation Sri Lanka*. **35**, 197-205 (2010).

Verhoyen, M. & Gofflot, A.. New outbreak in Belgium of tomato spotted wilt virus transmitted by thrips. New outbreak in Belgium of tomato spotted wilt virus transmitted by thrips. **55**, 1059-1068 (1990).

Zhang, Y. J., Wu, Q. J., Xu, B. Y. & Zhu, G. R. The occurrence and damage of *Frankliniella occidentalis* (Thysanoptera: Thripidae): a dangerous alien invasive pest in Beijing. *Chinese Plant Protect.* **4**, 58-59 (2003).
