# Supplementary material for: A global invasion by the thrip, Frankliniella occidentalis: Current virus vector status and its management
Source: Insect Sci. 2019 Oct 23;27(4):626–45. doi: 10.1111/1744-7917.12721 (PMC7318653; doi:10.1111/1744-7917.12721)
Supplement: Supplementary file 2 — Table S2. The worldwide distribution and host of viruses transmitted by Frankliniella occidentalis. [file INS-27-626-s002.doc]

Table S2 The worldwide distribution and host of viruses transmitted by *F. occidentalis*.

| **Virus** | **Region** | **Host** | **References** |
| --- | --- | --- | --- |
| Orthotospovirus |  |  |  |
| Alstroemeria necrotic streak orthotospovirus, AlNSV | Colombia | *Alstroemeria* sp. | Hassani-Mehraban *et al*. 2010 |
|  | Colombia | Tomato and bell pepper | Olaya *et al*. 2017; Gallo et al. 2019 |
| Chrysanthemum stem necrosis orthotospovirus, CSNV^a^ | Brazil | Tomato, *Datura stramonium* | Nagata *et al*. 2000, |
|  | Brazil | Tomato | Nagata *et al*. 1998, 2004 |
|  | Brazil | Chrysanthemum | Bezerra *et al*. 1999 |
|  | UK | Chrysanthemum | Mumford *et al*. 2003 |
|  | Korea | *Chrysanthemum morifolium* | Yoon *et al*. 2017a |
|  | Japan | Chrysanthemum | Okuda *et al*. 2013 |
|  | Japan | Chrysanthemum | Matsuura *et al*. 2007; Wu *et al*. 2015 |
| Groundnut ringspot orthotospovirus*, GRSV* | Argentina | Tomato,  *Nicotiana rustica* | Williams *et al*. 2001; Borbon *et al*. 2006 |
|  | Argentina, Córdoba | Peanut | de Breuil *et al*. 2007 |
|  | Argentina | Groundnut | Nacional *et al*. 1995 |
|  | Brazil | Tomato | Nagata *et al*. 2002, 2004 |
|  | Brazil | Coriander | Lima *et al*. 1999 |
|  | Brazil | Cocona | Boari *et al*. 2002 |
|  | Brazil | Cucumber | Spadotti *et al*. 2014 |
|  | Brazil | Cubiu | Boari *et al*. 2002 |
|  | Brazil | Peanut | Camelo-Garcia *et al*. 2014 |
|  | Brazil | Watermelon | Leao *et al*. 2015 |
|  | Ghana | Groundnut | Appiah *et al*. 2016 |
|  | The South Africa | Groundnut | Wijkamp *et al*. 1995 |
|  | The South Africa | Soybean | Pietersen *et al*. 2002 |
| GRSV-L_G_M_T_S_G_^*^ | USA, Florida | Tomato | Webster *et al*. 2010 |
|  | USA, Florida | Tomatillo，Eggplant，Pepper | Webster *et al*. 2011 |
|  | USA, South Carolina and New York | Tomato | Webster *et al*. 2015 |
| Impatiens necrotic spot orthotospovirus, INSV | USA | Lobelia | De Angelis *et al*. 1993 |
|  | USA | *Datura stramonium*, *Nicotiana benthamiana* | Hunter *et al*. 1995 |
|  | USA | Peanut | Pappu *et al*. 1999 |
|  | USA, Coastal California | Basil, Bell pepper,  Calla lily, Faba bean, Radicchio, Spinach, Lettuce | Kuo *et al*. 2014 |
|  | USA, Florida | Lisianthus | McGovern *et al*. 1997 |
|  | USA, California | Spinach | Liu *et al*. 2009 |
|  | USA, Georgia | Yellow Nutsedge (Cyperus esculentus), Purple Nutsedge | Martínez-Ochoa *et al*. 2004 |
|  | Bosnia and Herzegovina | Begonia | Trkulja *et al*. 2013a |
|  | China | *Phalaenopsis amabilis*, *Hymenocallis littoralis*, *Emilia sonchifolia* | Chen *et al*. 2013a |
|  | China, Yunnan | *Phalaenopsis, Dendrobium* | Zhang *et al*. 2010 |
|  | China | Pepper | Chen *et al*. 2016 |
|  | China | *Gentiana macrophylla* | Ding *et al*. 2011 |
|  | China | Spiderlily | Liu *et al*. 2010 |
|  | Hungary | Catharanthus roseus, Cyclamen persicum, Dendranthema × grandiflorum, Eustoma grandiflorum, Gerbera sp., Impatiens walleriana, Ocimum basilicum, and Verbena hybrida | Toth *et al*. 2007 |
|  | Egypt | *Impatiens* sp | El-Wahab *et al*. 2011 |
|  | Italy, Liguria | *Anemone*, *Ranunculus* | Vaira *et al*. 1993 |
|  | Italy | Tomato | Finetti *et al*. 2000 |
|  | Italy, Tuscany | *Spathiphyllum sp.* | Materazzi *et al*. 2001 |
|  | Italy, Emilia-Romagna | Lettuce, Pepper, Cucumber | Vicchi *et al*. 1999 |
|  | Iran | *Rosa sp., Gazania sp., Chrysanthemum sp., Leucanthemum sp., Matricaria camomila, Pelargonium roseum, Salvia sp., Dianthus caryophyllus, Gazania sp. and Bougainvillea spectabilis* | Shahraeen *et al*. 2002 |
|  | Japan | *Verbena*×*hybrida* *Impatiens* sp | Sakurai *et al*. 2004 |
|  | Japan | *Cerastium glomeratum*, *Cardamine scutata*, *Galiumspurium var .echinospermon*, *Stellaria aquatica*, *S. media*, *Veronica arvensis*, *V. persic* | Okuda *et al*. 2010 |
|  | Israel | *Anemone coronaria*, *Emilia* spp. | Gera *et al*. 1999 |
|  | the Netherlands | *Impatiens* sp | Wijkamp *et al*. 1993 |
|  | the Netherlands | *Impatiens* sp | Wijkamp *et al*. 1995 |
|  | the Czech Republic | *Stellaria media* | Mertelik *et al*. 2000 |
|  | New Zealand | freesia, impatiens, lobelia, primula，ranunculus，begonia, gerbera, cyclamen | Lebas *et al*., 2004, Pappu *et al*., 2009 |
|  | Mexico | Tomatillo, Pepper | González-Pacheco *et al*. 2013 |
|  | Spain | *Asplenium nidusavis* | Lavina *et al*. 1994 |
|  | Spain | *Tomato* | Haan *et al*. 1999 |
|  | Slovenia | Pepper, Chrysanthemum, *Impatiens walerana* | Mavric *et al*. 2001 |
|  | Portugal | Lettuce, Pepper, Tomato | Louro 1996 |
| Tomato chlorotic spot orthotospovirus, TCSV | Argentina | Sweet pepper, Potato, Celery, Lisianthus, Weeds, Tomato | Gracia *et al*. 1999; Dal Bio *et al*. 2001; Williams *et al*. 2001 |
|  | Brazil | Tomato | Nagata *et al*. 2004, |
|  | Brazil | Tomato | Wijkamp *et al*. 1995 |
|  | Brazil | Lettuce, endive, gilo | Colariccio *et al*. 2001a, b; Eiras *et al*. 2002; Rabelo *et al*. 2002 |
|  | Cuba | Tomato | Martinez-Zubiaur *et al*. 2016 |
|  | Cuba | Pepper and Common Bean | González-Alvarez *et al*. 2017 |
|  | Dominican Republic | Tomato | Batuman *et al*. 2014 |
|  | Dominican Republic | Long Beans, Chili Peppers | Almeida *et al*. 2014 |
|  | Haiti | Peanut | Adegbola *et al*. 2016 |
|  | Puerto Rico | Tomato, Pepper, jimsonweed (Datura stramonium), and Lettuce | Estévez de Jensen *et al*. 2013; Estévez de Jensen *et al*. 2014 |
|  | USA, Florida | *Catharanthus roseus* | Warfield *et al*. 2015 |
|  | USA, Florida | Tomato | Londono *et al*. 2012 |
|  | USA, Florida | *Solanum aethiopicum*,  *Solanum americanum* | Badillo-Vargas *et al*. 2016 |
|  | USA, Florida | Basil, Hoya, false Christmas cactus, Purslane, and Portulaca | Webster *et al*. 2015 |
| Tomato spotted wilt orthotospovirus, TSWV | Australia | Tomato, Pepper, Alstroemeria, Calendula, Chinese aster, Chrysanthemum, Cosmos, Dahlia, Delphinium, Gladiolus, Snapdragon, Statice, and Zinnia | Brittlebank 1919; Samuel *et al*. 1930; Best 1968; Latham *et al*. 1997; Sherman *et al*. 1998 |
|  | Australia | Common agapanthus | Wilson *et al*. 2000 |
|  | Argentina | Tomato,  *Nicotiana rustica* | Williams *et al*. 2001; Borbon *et al*. 2006 |
|  | Argentina, Mendoza | Potato | Salvalaggio *et al*. 2017 |
|  | Albania | Tomato, *Solanum nigrum*, *Stellaria media* | Cota *et al*. 2004 |
|  | Brazil | Tomato | Wijkamp *et al*. 1995 |
|  | Bulgaria | *Leuzea carthamoides* | Dikova *et al*. 2013 |
|  | Bulgaria | *Althaea officinalis*,  *Artemisia absinthium*, *Echinacea purpurea*, *purple coneflower*, *Foeniculum vulgare, Inula helenium, Leuzea carthamoides*,  *Nepeta cataria, Ocimum basilicum*, *Salvia officinalis*, *Salvia sclarea*, *Thymus vulgaris*, *Valeriana officinalis* | Dikova *et al*. 2011 |
|  | Bosnia and Herzegovina | Gloxinia | Trkulja *et al*. 2013b |
|  | Dominican Republic | Tomato and Pepper | Martínez *et al*. 2014 |
|  | Denmark | Bean | Van de Wetering *et al*. 1999 |
|  | Germany | *Capsicum annum* | Ogada *et al*. 2013, Shalileh *et al*. 2016 |
|  | Greece | Weeds, *Solanum*  *melongena*, *Celosia cristata*, *Dianthus chinensis*, *Stephanotis jloribunda*, *Catharanthus roseus* | Chatzivassiliou *et al*.1996, 1999, 2000, 2001, 2007 |
|  | China, Yunnan | Celery | Li *et al*. 2015 |
|  | China, Taiwan | Sweet Pepper | Zheng *et al*. 2010 |
|  | China, Yunnan | Cowpea | Xiao *et al*. 2016 |
|  | China, Yunnan | *Bidens pilosa* | Huang *et al*. 2016 |
|  | China | *Datura Stramonium, Capsicum annuum* | Zhao *et al*. 2016 |
|  | Chile | Lettuce | Rosales *et al*. 2007 |
|  | France | Bean | Van de Wetering *et al*. 1999 |
|  | France | pepper, tomato, eggplant, broad bean, lettuce, basil, chrysanthemum, aster, New Guinea impatiens, anemone and gloxinia | Marchoux *et al*. 1991 |
|  | Hungary | Pea | Salamon *et al*. 2012 |
|  | USA | *Nicotiana benthamiana*, *Emilia sonchifolia* | Sin *et al*. 2005 |
|  | USA | *Datura stramonium*, *Nicotiana benthamiana* | Hunter *et al*. 1995 |
|  | USA, Louisiana | *Ocimum basilicum*, *Melampodium divaricatum* | Holcomb *et al*. 1999, 2000 |
|  | USA, North Carolina | Tomato, Pepper, Tobacco, Cotton, *Stevia rebaudiana* | Eckel *et al*. 1996; Groves *et al*. 1998; Koehler *et al*. 2016 |
|  | USA, Georgia | Peanut, Pepper, Tomato, Tobacco, Tomatillo, Pinaceae, Vidalia Onion; Soybean | Culbreath *et al*. 1991; Diaz-Perez *et al*. 2000; Mandal *et al*. 2001, 2006; Mullis *et al*. 2004, 2006; Nischwitz *et al*. 2006a |
|  | USA, Kansas | *Datura stramonium Emilia sonchifolia* | Rotenberg *et al*. 2009 |
|  | USA, Florida | Tomato, Habanero and Tabasco Peppers | Momol *et al*. 2000, 2004 |
|  | USA | Chrysanthemum, Gloxinia, Leak | Van de Wetering *et al*. 1999; Nischwitz *et al*. 2006b |
|  | USA, Texas | Potato | Crosslin *et al*. 2009 |
|  | USA | Peanut | Garcia *et al*. 2000; Naidu *et al*. 2001; Shrestha *et al*. 2015; |
|  | USA | Ranunculus asiaticus | Whitfield *et al*. 2003 |
|  | Korea | Hot pepper, Bell pepper, Tomato | Ko *et al*. 2013 |
|  | Korea | *Brugmansia suaveolens* | Choi *et al*. 2014 |
|  | Korea | Paprika | Choi *et al*. 2004 |
|  | Korea | Potato | Choi and Choi 2015 |
|  | Korea | *Eustoma grandiflorum* | Yoon *et al*. 2017b |
|  | Kenya | Tomato | Wangai *et al*. 2001; Macharia *et al*. 2015 |
|  | Kenya | *Amaranthus hybridus*, *Solanum nigrum*, *Tagetes minuta*, *Datura stramonium* | Macharia *et al*. 2016 |
|  | the Netherlands | Petunia | Wijkamp *et al*. 1993 |
|  | the Netherlands | *Datura stramonium* | Nagata *et al*. 1999, Wijkamp *et al*. 1996a |
|  | the Netherlands | *Datura stramonium*, *Impatiens* sp, Tobacco | Wijkamp *et al*. 1996b |
|  | the Netherlands | Pepper | Kindt *et al*. 2003, Maris *et al*. 2003 |
|  | the Netherlands | Bean, Chrysanthemum, tomato, Cucumber | Van de Wetering *et al*. 1999 |
|  | Brazil | Tomato | Nagata *et al*. 2004 |
|  | UK | *Nicotiana rustica*; *Lycopersicon esculentum*; Chrysanthemum | Boonham *et al*. 2002 |
|  | Lebanon | Tomato | Abou-Jawdah *et al*. 2006 |
|  | Mexico | Chrysanthemum, Weeds | Martinez *et al*. 1999 |
|  | Mexico | Tomato | Holguín-Peña *et al*. 2007 |
|  | Montenegro | Pepper | Zindovic *et al*. 2011 |
|  | New Zealand | Egg plant | Van de Wetering *et al*. 1999 |
|  | New Zealand | Tomato | Chamberlain *et al*. 1936, 1938 |
|  | Tunisia | Tomato | Ben Moussa *et al*. 2000a |
|  | Turkey | Pepper, Lettuce, Tomato，  Squash | Yardimci *et al*. 2009; Sevik *et al*. 2012 |
|  | Turkey | Weed | Atakan *et al*. 2013 |
|  | Italy, Liguria | Pepper; tomato; Artichoke | Roggero *et al*. 2002 |
|  | Italy, Sardegna | Globe artichoke | Testa *et al*. 2011 |
|  | Italy | Celery and basil | Gallo *et al*. 1995 |
|  | Italy, Liguria | *Calendula officinaiis*,  *Capsicum annuum* | Vaira *et al*. 1993 |
|  | Italy | *Coprosma repens* | Polizzi *et al*. 2007 |
|  | Italy | *Arctotis* × *hybrida* | Parrella *et al*. 2013 |
|  | Italy | Bean | Van de Wetering *et al*. 1999 |
|  | Italy | Tomato | Finetti *et al*. 2000 |
|  | Italy, Sicily | Eggplant | Betti 1992 |
|  | Italy, Campania | tobacco | Carrieri *et al*. 2011 |
|  | Japan | Pepper | Abe *et al*. 2012, Inoue *et al*. 2004, Okazaki *et al*. 2007, 2011 |
|  | Japan | Chrysanthemum | Van de Wetering *et al*. 1999, Matsuura *et al*. 2002 |
|  | Jordan | Tomato | Anfoka *et al*. 2006 |
|  | India | Chrysanthemum | Renukadevi *et al*. 2015 |
|  | India | Sunflower | Subbaiah *et al*. 2000 |
|  | Israel | *Datura stramonium Emilia sonchifolia* | Kritzman *et al*. 2002 |
|  | Israel | Strawberry, Mango | Van de Wetering *et al*. 1999 |
|  | Israel | *Pittosporum tobira* | Gera *et al*. 2000a |
|  | Iran | Potato | Pourrahim *et al*. 2001 |
|  | Iran | Cucumber, Watermelon, Melon, Tomato | Massumi *et al*. 2007, 2009 |
|  | Iran | Soybean | Golnaraghi *et al*. 2001 |
|  | Saudi Arabia | Letture | Al-Shahwan *et al*. 2014 |
|  | South Africa | *Amaranthus thunbergii* | Kisten *et al*. 2016 |
|  | South Africa | Tobacco | Moore 1933 |
|  | South Africa | Tomato, pepper and potato | Moore *et al*. 1939 |
|  | Spain | Letture | Moreno *et al*. 2004 |
|  | Spain | Tomato | Aramburu *et al*. 1996 |
|  | Spain | *Diplotaxis erucoides* (L.) DC., *Beta maritima* L., *Phragmites communis* Trin., *Malva sylvestris* L., *Sonchus arvensis* L., *Sorghum halepense* L., *Panicum repens* L., *Atriplex patula* L., *Coronopus squamatus* (Forssk.) Ascherson, *Cuscuta* sp., *Xanthium spinosum* L., *Suaeda vera* J.F., and *Ecballium elaterium* | Jordá *et al*. 2000 |
|  | Spain | Papper, Tomato, Letture, Pea | Roca *et al*. 1997 |
|  | Slovenia | Pepper, Chrysanthemum, *Impatiens walerana* | Mavric *et al*. 2001 |
|  | Serbia | *Gerbera hybrid* | Stanković *et al*. 2011 |
|  | Serbia | Onion, garlic and chrysanthemum | Stanković *et al*. 2012, 2013 |
|  | Serbia | *Brugmansia sp* | Nikolić *et al*. 2013 |
|  | Portugal | Lettuce, Pepper, Tomato | Louro 1996 |
|  | Venezuela | Gerbera; Chrysanthemun; Pepper | Marys *et al*. 2014; Perez-Colmenares *et al*. 2015 |
|  | Zimbabwe | Potato, Eggplant, Pepper, Cherry pepper, Tomato, Butternut Squash; Chrysanthemum, Cucumber, Butternut | Karavina *et al*. 2016a, b; Karavina *et al*. 2017 |
| Tomato yellow ring virus, TYRV | China | Tomato | Chen *et al*. 2013b |
|  | Iran | Tomato, Cineraria, Chrysanthemum, Gazania, Potato, Soybean | Mortazavi *et al*. 2013; Mortazavi *et al*. 2015; Hassani-Mehraban *et al*. 2007; Golnaraghi *et al*. 2007; Rasoulpour *et al*. 2007 |
|  | Kenya | Tomato | Birithia *et al*. 2012 |
|  | Poland | Tomato | Zarzyńska-Nowak*et al*. 2016 |
| Tomato zonate spot orthotospovirus, TZSV | China | *Crinum asiaticum* | Wu *et al*. 2016 |
|  | China | Tomato, Pepper Chili (*Capsicum annuum*  L.), Weeds (*Bidens pilosa* and *Rumex dentatus*) | Dong *et al*. 2008, 2010; Zheng *et al*. 2014 |
|  | China | *Iris tectorum* | Liu *et al*. 2015b |
|  | China | Tobacco | Cai *et al*. 2011 |
|  | China | Potato | Huang *et al*. 2015 |
| *Ilarvirus* |  |  |  |
| Parietaria mottle virus, PMoV | Spain | Tomato, Pepper | Aramburu *et al*. 2001 |
|  | France | Tomato | Ramasso *et al*. 1997 |
|  | Italy | *Parietaria officinalis*, *Chenopodium quinoa* | Caciagli *et al*. 1989; Scott *et al*. 2006 |
|  | Italy | *Mirabilis jalapa* | Parrella. 2002 |
|  | Italy | Tomato | Roggero *et al*. 2000 |
|  | Italy | *Capsicum annuum* | Parrella *et al*. 2016 |
|  | Italy | *Diplotaxis tenuifolia* | Parrella *et al*. 2017 |
|  | Spain | *Capsicum annuum* | Janssen *et al*. 2005 |
|  | Spain | Parietaria, Tomato | Galipienso *et al*. 2005, 2008, 2009 |
|  | Greece | Tomato | Roggero *et al*. 2000 |
| *Alphacarmovirus* |  |  |  |
| Pelargonium flower break virus, PFBV | China | Pelargonium | Wei *et al*. 2015 |
|  | Germany | Pelargonium | Krczal *et al*. 1995 |
|  | The Netherlands | Pelargonium | Bouwen *et al*. 1992 |
|  | UK | Pelargonium | Stone *et al*. 1973 |
|  | Spain | Geraniums (Pelargonium × hortorum Bailey) | Ivars. *et al*. 2004 |
|  | Spain | Pelargonium × hortorum; Pelargonium zonale; Chenopodium quinoa | Rico. *et al*. 2004, 2006 |
| *Machlomovirus* |  |  |  |
| Maize chlorotic mottle virus, MCMV | Argentina | Maize | Teyssandier *et al*. 1981 |
|  | China | Maize | Xie *et al*. 2011; Zhao *et al*. 2014; Chen *et al*. 2017 |
|  | China | Sorghum, Coix Seed | Huang *et al*. 2016 |
|  | China, Taiwan | Maize | Deng *et al*. 2014 |
|  | Colombia | Maize | Morales *et al*. 1999 |
|  | Congo | Maize | Lukanda *et al*. 2014 |
|  | Ecuador | Maize | Quito-Avila *et al*. 2016 |
|  | Peru | Maize | Castillo-Loayza 1977 |
|  | Mexia | Maize | Carrera-Martinez *et al*. 1989 |
|  | USA | Maize | Jiang *et al*. 1990; Niblett *et al*. 1978 |
|  | Spain | *Sorghum halepense*, Maize | Achon *et al*. 2017 |
|  | Kenya | Maize | Wangai *et al*. 2012 |
|  | Kenya | Finger Millet | Kusia *et al*. 2015 |
|  | Rwanda | Maize | Adams *et al*. 2014 |
|  | Tanzania | Maize | Mahuku *et al*. 2015a |
|  | Thailand | Maize | Klingkong *et al*. 1982 |
|  | Uganda | Maize | Mahuku *et al*. 2015a |
|  | Ethiopia | Maize | Mahuku *et al*. 2015b |

* A reassortant isolate GRSV-L_G_M_T_S_G_, composed of the L and S RNAs from GRSV and the M RNA from TCSV, was reported from tomatoes in Florida in 2010 (Webster et al. 2011).

**References**

Abe, H. *et al.* Antagonistic Plant Defense System Regulated by Phytohormones Assists Interactions Among Vector Insect, Thrips and a Tospovirus. *Plant Cell Physiol*. **53**, 204–212 (2012)..

Abou-Jawdah, Y., El Mohtar, C., Sobh, H. & Nakhla, M. K. First Report of *Tomato spotted wilt virus* on Tomatoes in Lebanon. *Plant Dis*. **90**, 376–376 (2006)..

Achon, M.A., Serrano, L., Clemente-Orta, G. & Sossai, S First Report of *Maize chlorotic mottle virus* on a Perennial Host, *Sorghum halepense* , and Maize in Spain. *Plant Dis*. **101**, 393–393 (2017)..

Adams, I.P. *et al.* First report of maize lethal necrosis disease in Rwanda. *New Dis Reports*, 29, 22.

Adegbola, R.O., Fulmer, A.M., Williams, B., Brenneman, T.B., Kemerait, R.C., Sheard, W., *et al.* (2016). First Report of the Natural Occurrence of *Tomato chlorotic spot virus* in Peanuts in Haiti. *Plant Dis*. **100**, 1797 (2014)..

Almeida, M.M.S. *et al.* The First Report of *Tomato chlorotic spot virus* (TCSV) Infecting Long Beans and Chili Peppers in the Dominican Republic. *Plant Dis*. **98**, 1285–1285 (2014)..

Anfoka, G. H., Abhary, M. & Stevens, M. R. Occurrence of *Tomato spotted wilt virus* (TSWV) in Jordan. *EPPO Bull*. **36**, 517–522 (2006).

Appiah, A.S., Offei, S.K., Tegg, R.S. & Wilson, C.R. Varietal Response to Groundnut Rosette Disease and the First Report of *Groundnut ringspot virus* in Ghana. *Plant Dis*. **100**, 946–952 (2016).

Aramburu, J. First Report of *Parietaria mottle virus* on Tomato in Spain. *Plant Dis*. **85**, 1210 (2001).

Aramburu, J., Riudavets, J., Arno, J., Lavina, A. & Moriones, E. Rapid serological detection of tomato spotted wilt virus in individual thrips by squash-blot assay for use in epidemiological studies. *Plant Pathol*. **45**, 367–374 (1996).

Atakan, E., Kamberoğlu, M.A. & Uygur, S. Role of weed hosts and the western flower thrips, *Frankliniella occidentalis*, in epidemiology of *Tomato spotted wilt virus* in the Çukurova region of Turkey. *Phytoparasitica* **41**, 577–590 (2013).

Batuman, O., Rojas, M., Almanzar, A. & Gilbertson, R. First report of Tomato chlorotic spot virus in processing tomatoes in the Dominican Republic. *Plant Dis*, 98, 286.

Betti, L. (1992). Tomato spotted wilt tospovirus on eggplant in Sicily. Phytopathol. Mediterr. **31**, 119–120 (2014).

Best, R. J. Tomato spotted wilt virus. Advances in Virus Research **13**, 65-145 (1968).

Bezerra, I.C., de O. Resende, R., Pozzer, L., Nagata, T., Kormelink, R. & De Ávila, A.C. Increase of tospoviral diversity in brazil with the identification of two new tospovirus species, one from chrysanthemum and one from zucchini. *Phytopathology* **89**, 823–830 (1999).

Birithia, R., Subramanian, S., Villinger, J., Muthomi, J.W., Narla, R.D. & Pappu, H.R. First Report of *Tomato yellow ring virus* (*Tospovirus* , *Bunyaviridae*) Infecting Tomato in Kenya. *Plant Dis*. **96**, 1384–1384 (2012).

Boari, A.J. *et al.* Detection and partial characterization of an isolate of *Groundnut ringspot virus* in *Solanum sessiliflorum*. *Fitopatol Bras*, **27**, 249–253 (2002).

Boonham, N. *et al.* The detection of *Tomato spotted wilt virus* (TSWV) in individual thrips using real time fluorescent RT-PCR (TaqMan). *J Virol Methods* **101**, 37–48 (2002).

Borbon, C.M., Gracia, O. & Piccolo, R. Relationships between Tospovirus Incidence and Thrips Populations on Tomato in Mendoza, Argentina. *J Phytopathol*. **154**, 93–99 (2006).

Bouwen, I. & Maat, D.Z. Pelargonium flower-break and pelargonium line pattern viruses in the Netherlands; purification, antiserum preparation, serological identification, and detection in pelargonium by ELISA. *Netherlands J Plant Pathol*. **98**, 141–156 (1992).

Breuil, S., Abad, J.A., Nome, C.F., Giolitti, F.J., Lambertini, P.L. & Lenardon, S. Groundnut ringspot virus: An emerging Tospovirus inducing disease in peanut crops. *J Phytopathol*, **155**, 251–254 (2007).

Brittlebank, C. C. Tomato diseases. Journal of Agriculture, Victoria 27, 231(1919).

Caciagli, P., Boccardo, G. & Lovisolo, O. Parietaria mottle virus, a possible new ilarvirus from *Parietaria officinalis* (Urticaceae). Plant Pathol. 38, 577–584(1989).

Cai, J.H. *et al.* Molecular Identification and Characterization of Tomato zonate spot virus in Tobacco in Guangxi, China. *Plant Dis*. **95**, 1483–1483 (2011).

Camelo-Garca, V.M., Lima, lison Fabricio B, Mansilla-C??rdova, P.J., Rezende, J.A.M., Kitajima, E.W. & Barreto, M. Occurrence of Groundnut ringspot virus on Brazilian peanut crops. *J Gen Plant Pathol*. **80**, 282–286 (2014).

Carrera-Martinez, H., Lozoya-Saldana, H., Mendoza-Zamora, C. & Alvizo-Villasana, H. lmmunoabsorcion enzimatica (ELISA) en Ia identificaciony distribucion del virus moteado clorotico del maiz (VMCM) en el estado de Mexico. *Rev. Mex. Fitopatol*. **7**, 20–25 (1989).

Carrieri, R., Sorrentino, R., Lahoz, E. & Alioto, D. First Report of *Tomato spotted wilt virus* on Tobacco in Campania, Italy. *Plant Dis*. **95**, 611–611 (2011).

Castillo-Loayza, J. Maize virus and virus-like diseases in Peru. In: *Proc Int Maize Virus Disease Colloquim Workshop* (eds. Williams, L., Gordon, D. & Nault, L.). pp. 40–44 (1977).

Chamberlain, E.. & Taylor, G. The occurrence of spotted-wilt on tomatoes in New Zealand. *N. Z. J. Agric*. **52**, 9–17 (1936).

Chamberlain, E. & Taylor, G. Spotted wilt. Host range and transmission by thrips. *N. Z. J. Sci. Tech. Sect. A*. **20**, 133–142 (1938).

Chatzivassiliou, EK Livieratos, I., Jenser, G. & Katis, N. Ornamental plants and thrips populations associated with tomato spotted wilt virus in Greece. *Phytoparasitica* **28**, 257–264 (2000).

Chatzivassiliou, E.K. *et al.* Weeds in Greenhouses and Tobacco Fields Are Differentially Infected by Tomato spotted wilt virus and Infested by Its Vector Species. *Plant Dis*. **85**, 40–46 (2001).

Chatzivassiliou, E.K., Peters, D. & Lolas, P.. Occurrence of *Tomato spotted wilt virus* in *Stevia rebaudiana* and *Solanum tuberosum* in Northern Greece. *Plant Dis*. **91**, 1205–1205 (2007).

Chatzivassiliou, Nagata, Katis & Peters. Transmission of tomato spotted wilt tospovirus by Thrips tabaci populations originating from leek. *Plant Pathol*. **48**, 700–706 (1999)..

Chatzivassilou, EK Livieratos, I., Katis, N., Avegelis, A. & Lykouressis, D. Occurrence of tomato spotted wilt virus in vegetables and ornamentals in Greece. *Acta Hortic*. **431**, 44–50 (1996).

Chen, L. *et al.* One-step reverse transcription loop-mediated isothermal amplification for the detection of *Maize chlorotic mottle virus* in maize. *J Virol Methods* **240**, 49–53 (2017).

Chen, Q. & Wei, T. Viral receptors of the gut: insect-borne propagative plant viruses of agricultural importance. *Curr Opin Insect Sci*. **16**, 9–13 (2016).

Chen, T.C., Li, J.T., Fan, Y.S., Yeh, Y.C., Yeh, S.D. & Kormelink, R. Molecular characterization of the full-length L and M RNAs of *Tomato yellow ring virus*, a member of the genus *Tospovirus*. *Virus Genes* **46**, 487–495 (2013a).

Chen, X., Xu, X., Li, Y. & Liu, Y. Development of a real-time fluorescent quantitative PCR assay for detection of Impatiens necrotic spot virus. *J Virol Methods* **189**, 299–304 (2013b).

Choi, G.-S., Kim, J.S., Choi, J.K. & Kim, J.H. Characterization of *Tomato spotted wilt virus* from Paprika in Korea. *Plant Pathol J*. **20**, 297–301 (2004).

Choi, S.K., Cho, I.S., Choi, G.S. & Yoon, J.Y. First Report of *Tomato spotted wilt virus* in *Brugmansia suaveolens* in Korea. *Plant Dis*. **98**, 1283–1283 (2014).

Choi, S.K. & Choi, G.S. First Report of *Tomato spotted wilt virus* in *Solanum tuberosum* in Korea. *Plant Dis*. **99**, 1657 (2015).

Colariccio, A., Chaves, A., Eiras, M. & Chagas, C. Identification of the tomato chlorotic spot virus in endive (*Cichorium endiva* L.). *Summa Phytopathol*. **27**, 325–327 (2001a).

Colariccio, A., Eiras, M., Alexandre, L., Chaves, R. & Chagas, C. Characterization of *Tomato chlorotic spot virus* from hydroponic grown lettuce in Brazil. In: *Thrips and Tospoviruses: Proceedings of the 7th International Symposium on Thysanoptera* (eds. Marullo, R. & Mound, L.). ANIC, Reggio Calabria, Italy, pp. 99–104 (2001b).

Cota, E. & Merkuri, J. Introduction of *Frankliniella occidentalis* and occurrence of *Tomato spotted wilt tospovirus* in Albania. *EPPO Bull*. **34**, 421–422 (2004).

Crosslin, J.M., Mallik, I. & Gudmestad, N.C. First Report of *Tomato spotted wilt virus* Causing Potato Tuber Necrosis in Texas. *Plant Dis*. **93**, 845–845 (2009).

Culbreath, A.K., Csinos, A.S., Bertrand, P.F. & Demski, J.W. Tomato Spotted Wilt Virus Epidemic in Flue-Cured Tobacco in Georgia. *Plant Dis*. (1991)

Dal Bio, E., Chiarrone, G., Rolleri, J. & Ronc, L. New tospoviruses found in La Plata. *Rev la Fac Agron (La Plata)*, **104**, 35–40 (2001).

DeAngelis, J. D., Sether, D. M. & Rossignol, P.A. Survival, development and reproduction in western flower thrips (Thysanptera: Thripidae) exposed to impatiens necrotic spot virus. Environmental Entomology **22**, 1308–1312 (1993).

Deng, T.C., Chou, C.M., Chen, C.T., Tsai, C.H. & Lin, F.C. First Report of *Maize chlorotic mottle virus* on Sweet Corn in Taiwan. *Plant Dis*. **98**, 1748–1748 (2014).

Díaz-Pérez, J.C. & Pappu, H.R. First Report of *Tomato spotted wilt virus* Infection of Tomatillo in Georgia. *Plant Dis*. **84**, 1155–1155 (2000).

Dikova, B.. *Tomato spotted wilt virus* on some medicinal and essential oil-bearing plants in Bulgaria. Bulg. J. Agric. Sci. **17**, 306-313 (2011).

Dikova, B., Petrov, N., Djourmanski, A. & Lambev, H. First Report of *Tomato spotted wilt virus* on a New Host *Leuzea carthamoides* in Bulgaria and the World. *Plant Dis*. **97**, 1258–1258 (2013).

Ding, M., Yin, Y., Fang, Q., Li, S. & Zhang, Z. First Report of *Impatiens necrotic spot virus* in *Gentiana macrophylla* in China. *Plant Dis*. **95**, 357–357 (2011).

Dong, J. H. *et al.* Characterization of tomato zonate spot virus, a new tospovirus in China. *Arch Virol*. **153**, 855–64 (2008).

Dong, J., Zhang, Z., Yin, Y., Cheng, X., Ding, M. & Fang, Q. Natural host ranges of *Tomato zonate spot virus* in Yunnan. *J. Insect Sci*. **10**, 12–13 (2010).

Eckel, C.S., Cho, K., Walgenbach, J.F., Kennedy, G.G. & Moyer, J.W. Variation in thrips species composition in field crops and implications for tomato spotted wilt epidemiology in North Carolina. *Entomol. Exp. Appl*. **78**, 19–29 (1996).

El-Wahab, A. S. E. D. A., El-Sheikh, M. A. K. & Elnagar, S. First record of *Frankliniella occidentalis* and *Impatiens necrotic spot virus* in Egypt. J. Life Sci. **5**, 690–696 (2011).

Eiras, M., Chaves, A., Colariccio, A., Harakava, R., de Araujo, J. & Chagas, C. Characterization of a *Tomato chlorotic spot virus* isolated from gilo in Peraiba Valley, Sao Paulo, Brazil. *Fitopatol Bras*. **27**, 285–291 (2002).

Estévez de Jensen, C. & Adkins, S. First report of *Tomato chlorotic spot virus* in lettuce in Puerto Rico. *Plant Dis*. 98, 1015 (2014).

Estévez de Jensen, C. *et al.* First Report of *Tomato chlorotic spot virus* (TCSV) in Tomato, Pepper, and Jimsonweed in Puerto Rico. *Plant Heal Prog* (2013a).

Finetti Sialer, M.M. & Gallitelli, D. The occurrence of *Impatiens necrotic spot virus* and *Tomato spotted wilt virus* in mixed infection in tomato. *J. Plant Pathol*. **82**, 244 (2000)..

Galipienso, L., Del Carmen Herranz, M., López, C., Pallás, V. & Aramburu, J. Sequence analysis within the RNA 3 of seven Spanish tomato isolates of *Parietaria mottle virus* (PMoV-T) reveals important structural differences with the parietaria isolates (PMoV). *Eur. J. Plant Pathol*. **120**, 125–135 (2008)..

Galipienso, L., Herranz, M.C., Pallás, V. & Aramburu, J. Detection of a tomato strain of *Parietaria mottle virus* (PMoV-T) by molecular hybridization RT-PCR in field samples from north-eastern Spain. *Plant Pathol.* **54**, 29–35 (2005).

Galipienso, L., Rubio, L., López, C., Soler, S. & Aramburu, J. Complete nucleotide sequence of a Spanish isolate of *Parietaria mottle virus* infecting tomato. *Virus Genes* **39**, 256–260 (2009).

Gallo, S., Gotta, P. & Lisa, V. Tomato spotted wilt virus. Piemonte Agric. **19** (Suppl 1):1–8 (in Italian) (1995).

Gallo, Y., Sierra, A., Muñoz, L. Marín, M. & Gutiérrez, P. Genome characterization of three Alstroemeria necrotic streak orthotospovirus (ANSV) isolates naturally infecting bell pepper (*Capsicum annuum*) in Antioquia (Colombia). *Trop. plant pathol.* On line (2019).

Garcia, L.E., Brandenburg, R.L. & Bailey, J.E. Incidence of *Tomato spotted wilt virus* (*Bunyaviridae*) and Tobacco Thrips in Virginia-Type Peanuts in North Carolina. *Plant Dis*. **84**, 459–464 (2000).

Gera, A., Kritzman, A. & Cohen, J. *Pittosporum tobira* : A New Host for *Tomato spotted wilt virus*. *Plant Dis*. **84**, 491–491 (2000).

Gera, A., Kritzman, A., Cohen, J. & Raccah, B. First Report of Impatiens Necrotic Spot Tospovirus (INSV) in Israel. *Plant Dis*. **83**, 587–587(1999)..

Gilbertson, R.L., Batuman, O., Webster, C.G. & Adkins, S. Role of the Insect Supervectors *Bemisia tabaci* and *Frankliniella occidentalis* in the Emergence and Global Spread of Plant Viruses. *Annu. Rev. Virol*. **2**, 67–93 (2015).

Gitaitis, R.D., Dowler, C.C. & Chalfant, R.B. Epidemiology of Tomato Spotted Wilt in Pepper and Tomato in Southern Georgia. *Plant Dis*. **82**, 752–756 (1998).

Golnaraghi, A.R., Pourrahim, R., Farzadfar, S., Ohshima, K., Shahraeen, N. & Ahoonmanesh, A. Incidence and distribution of tomato yellow fruit ring virus on soybean in Iran. *Plant Pathol J*. (2007).

Golnaraghi, A.R., Shahraeen, N., Pourrahim, R., Ghorbani, S. & Farzadfar, S. First Report of *Tomato spotted wilt virus* on Soybean in Iran. *Plant Dis*. **85**, 1290–1290 (2001).

González-Alvarez, H., Chang-Sidorchuk, L., Barboza Vargas, N., González Arias, G. & Martínez-Zubiaur, Y. First Report of *Tomato chlorotic spot virus* infecting Pepper and Common Bean in Cuba. *Plant Dis*. **101**, 1064 (2017).

González-Pacheco, B.E. & Silva-Rosales, L. First Report of *Impatiens necrotic spot virus* in Mexico in Tomatillo and Pepper Plants. *Plant Dis*. **97**, 1124–1124 (2013).

Gracia, O., De Borbon, C.M., Granval De Millan, N. & Cuesta, G. V. Occurrence of different tospoviruses in vegetable crops in Argentina. *J. Phytopathol*. **147**, 223–227 (1999a).

Groves, R.L., Kennedy, G.G., Walgenbach, J.F. & Moyer, J.W. Inoculation of Tomato Spotted Wilt Virus into Cotton. *Plant Dis*. **82**, 959–959 (1998).

Haan, P. *et al.* The nucleotide sequence of the S RNA of *Impatiens* necrotic spot virus, a novel tospovirus. *FEBS Lett*. **306**, 27–32 (1992).

Hassani-Mehraban, A. *et al.* A distinct tospovirus causing necrotic streak on *Alstroemeria* sp. in Colombia. *Arch Virol*. **155**, 423–8 (2010).

Hassani-Mehraban, A., Saaijer, J., Peters, D., Goldbach, R. & Kormelink, R. Molecular and biological comparison of two tomato yellow ring virus (TYRV) isolates: Challenging the Tospovirus species concept. *Arch Virol*. **152**, 85–96 (2007).

Holcomb, G.E. & Valverde, R.A. First Report of *Oidium* sp. Powdery Mildew and *Tomato spotted wilt virus* on *Melampodium divaricatum*. *Plant Dis*. **84**, 1152–1152 (2000).

Holcomb, G.E., Valverde, R.A., Sim, J. & Nuss, J. First Report on Natural Occurrence of Tomato Spotted Wilt Tospovirus in Basil (*Ocimum basilicum*). *Plant Dis*. **83**, 966–966 (1999).

Holguín-Peña, R.J. & Rueda-Puente, E.O. Detection of *Tomato spotted wilt virus* in Tomato in the Baja California Peninsula of Mexico. *Plant Dis*. **91**, 1682–1682 (2007).

Huang, C. J., Liu, Y., Yu, H. Q. & Li, B. L. Occurrence of *Tomato zonate spot virus* on Potato in China. *Plant Dis*. **99**, 733–733 (2015).

Huang, C. J., Liu, Y., Yu, H. Q., Liu, B .Z. & Qing, L. Bidens pilosa is a Natural Host of *Tomato spotted wilt virus* in Yunnan Province, China. *Plant Dis*. **100**, 1957–1957 (2016).

Huang, J., Wen, G. S., Li, M. J., Sun, C. C., Sun, Y., Zhao, M. F., and He, Y. Q. First report of *Maize chlorotic mottle virus* naturally infecting sorghum and coix seed in China. Plant Dis. **100**, 1955 (2016).

Hunter, W.B., H.T. Hsu, R.H.L. A novel method for tospovirus acquisition by thrips. *Phytopathol.* **85**, 480–483 (1995).

Inoue, T., Sakurai, T., Murai, T. & Maeda, T. Specificity of accumulation and transmission of tomato spotted wilt virus (TSWV) in two genera, Frankliniella and Thrips (Thysanoptera: Thripidae). *Bull Entomol Res*. **94**, 501–507 (2004).

Ivars, P., Alonso, M., Borja, M. & Hernández, C. Development of a non-radioactive dot-blot hybridisation assay for the detection of *Pelargonium flower break virus* and P*elargonium line pattern virus*. *Eur. J. Plant Pathol*. **110**, 275–283 (2004a).

Janssen, D., Saez, E., Segundo, E., Martín, G., Gil, F. & Cuadrado, I.M. *Capsicum annuum* - A new host of Parietaria mottle virus in Spain. *Plant Pathol*. **54**, 567 (2005).

Jiang, X., Wilkinson, D. & Berry, J. An outbreak of maize chlorotic mottle virus in Hawaii and possible association with thrips. *Phytopathol.* **80**, 1060 (1990).

Jordá, C., Font, I., Lázaro, A., Juarez, M., Ortega, A. & Lacasa, A. New Natural Hosts of *Tomato spotted wilt virus*. *Plant Dis*. **84**, 489–489 (2000).

Karavina, C. & Gubba, A. Detection and characterization of Tomato spotted wilt virus infecting field and greenhouse-grown crops in Zimbabwe. *Eur. J. Plant Pathol*. 1915 (2017).

Karavina, C., Ibaba, J.D. & Gubba, A. First Report of *Tomato spotted wilt virus* Infecting Butternut Squash (*Cucurbita moschata*) in Zimbabwe. *Plant Dis*. **100**, 870–870 (2016a).

Karavina, C., Ximba, S., Ibaba, J.D. & Gubba, A. First Report of a Mixed Infection of *Potato virus Y* and *Tomato spotted wilt virus* on Pepper (*Capsicum annuum*) in Zimbabwe. *Plant Dis*. **100**, 1513–1513 (2016b).

Kindt, F., Joosten, N.N., Peters, D. & Tjallingii, W.F. Characterisation of the feeding behaviour of western flower thrips in terms of electrical penetration graph (EPG) waveforms. *J Insect Physiol*. **49**, 183–191 (2003).

Kisten, L., Moodley, V., Gubba, A. & Mafongoya, P.L. First Detection of *Tomato spotted wilt virus* (TSWV) on *Amaranthus thunbergii* in South Africa. *Plant Dis*. **100**, 2176–2176 (2016).

Klingkong, T. & Sutabutra, T. A new virus disease of maize in Thailand. In: *Proc. Int. Maize Virus Dis. Colloq. Workshop.* (eds. Gordon, D.T., Knoke, J.K., Nault, R. & Ritter, R.). OARDC, Wooster, OH., pp. 191–193 (1982).

Ko, S. *et al.* Pattern of the Occurrence of Tomato spotted wilt virus in Jeonnam Province. *Res Plant Dis*. **19**, 273–280 (2013).

Koehler, A.M., Brown, J.A., Huber, B., Wehner, T.C. & Shew, H.D. First Report of *Tomato spotted wilt virus* in *Stevia rebaudiana* in North Carolina. *Plant Dis*. **100**, 1251–1251 (2016).

Krczal, G. *et al.* Transmission of pelargonium flower break virus (PFBV) in irrigation systems and by thrips. *Plant Dis*. (1995).

Kritzman, A., Gera, A., Raccah, B., van Lent, J. W. M. & Peters, D. The route of tomato spotted wilt virus inside the thrips body in relation to transmission efficiency. *Arch Virol*. **147**, 2143–2156 (2002).

Kuo, Y.W., Gilbertson, R.L., Turini, T., Brennan, E.B., Smith, R.F. & Koike, S.T. Characterization and Epidemiology of Outbreaks of *Impatiens necrotic spot virus* on Lettuce in Coastal California. *Plant Dis*. **98**, 1050–1059 (2014).

Kusia, E.S. *et al.* First Report of Lethal Necrosis Disease Associated With Co-Infection of Finger Millet With *Maize chlorotic mottle virus* and *Sugarcane mosaic virus* in Kenya. *Plant Dis*. **99**, 899 (2015).

Latham, L. J. & Jones, R. A. C. Occurrence of tomato spotted wilt tospovirus in native flora, weeds, and horticultural crops. *Aust. J. Agric. Res*. **48**, 359 (1997).

Lavina A., & Battle A. First report of impatiens necrotic spot virus in *Asplenium nidusavis* in Spain. Plant Dis. **78**, 316 (1994).

Leão, E.U. *et al.* *Citrullus lanatus* is a New Natural Host of Groundnut ringspot virus in Brazil. *J. Phytopathol*. **163**, 1014–1018 (2015).

Lebas, B.S., Ochoa-Corona, F., Elliott, D., Tang, Z., Alexander, B.J. & Froud, K.J. An investigation of an outbreak of Impatiens necrotic spot virus in New Zealand. *Phytopathol.* **94**, S57–58 (Abstr.) (2004).

Li, Y.Y., Xiao, L., Tan, G.L., Fu, X.P., Li, R.H. & Li, F. First Report of *Tomato spotted wilt virus* on Celery in China. *Plant Dis*. **99**, 734 (2015).

Lima, M.F., de Ávila, A.C., da G. Wanderley, L.J., Nagata, T. & da Gama, L.J.W. Coriander: A New Natural Host of Groundnut Ring Spot Virus in Brazil. *Plant Dis*. **83**, 878–878 (1999).

Liu, H.-Y., Sears, J.L. & Mou, B. Spinach (*Spinacia oleracea*) is a New Natural Host of *Impatiens necrotic spot virus* in California. *Plant Dis*. **93**, 673–673 (2009).

Liu, Y., Huang, C.J., Tao, X.R. & Yu, H.Q. First Report of *Tomato zonate spot virus* in *Iris tectorum* in China. *Plant Dis*. **99**, 164–164 (2015b).

Liu, Y.T., Zheng, Y.X., Li, Y.Z. & Li, Z.Y. First Report of *Impatiens necrotic spot virus* on Spiderlily in China. *Plant Dis*. **94**, 484–484 (2010).

Londoño, A., Capobianco, H., Zhang, S. & Polston, J.E. First record of *Tomato chlorotic spot virus* in the USA. *Trop Plant Pathol*. **37**, 333–338 (2012).

Louro, D. Detection and identification of tomato spotted wilt virus and impatiens necrotic spot virus in Portugal. *Acta Hortic*. **431**, 99–105 (1996).

Lukanda, M. *et al.* First Report of *Maize chlorotic mottle virus* Infecting Maize in the Democratic Republic of the Congo. *Plant Dis*. **98**, 1448–1448 (2014).

Macharia, I. *et al.* Diversity of Thrips Species and Vectors of Tomato Spotted Wilt Virus in Tomato Production Systems in Kenya. *J. Econ. Entomol*. **108**, 20–28 (2015).

Macharia, I., Backhouse, D., Wu, S.-B. & Ateka, E.M. Weed species in tomato production and their role as alternate hosts of Tomato spotted wilt virus and its vector Frankliniella occidentalis. *Ann. Appl. Biol*. **169**, 224–235 (2016).

Mahuku, G. *et al.* Maize Lethal Necrosis (MLN), an Emerging Threat to Maize-Based Food Security in Sub-Saharan Africa. *Phytopathol.***105**, 956–965 (2015a).

Mahuku, G. *et al.* First Report of *Maize chlorotic mottle virus* and Maize Lethal Necrosis on Maize in Ethiopia. *Plant Dis*. **99**, 1870–1870 (2015b).

Mandal, B., Pappu, H.R., Csinos, A.S. & Culbreath, A.K. Response of Peanut, Pepper, Tobacco, and Tomato Cultivars to Two Biologically Distinct Isolates of *Tomato spotted wilt virus*. *Plant Dis*. **90**, 1150–1155 (2006).

Mandal, B., Pappu, H.R. & Culbreath, A.K. Factors Affecting Mechanical Transmission of *Tomato spotted wilt virus* to Peanut (*Arachis hypogaea* ). *Plant Dis*. **85**, 1259–1263 (2001).

Marchoux, G., Gebre-Selassie, K. & Villevielle, M. Detection of tomato spotted wilt virus and transmission by Frankliniella occidentalis in France. *Plant Pathol*. **40**, 347–351 (1991).

Maris, P.C., Joosten, N.N., Peters, D. & Goldbach, R.W. Thrips Resistance in Pepper and Its Consequences for the Acquisition and Inoculation of Tomato spotted wilt virus by the Western Flower Thrips. *Phytopathol.* **93**, 96–101 (2003).

Martínez-Ochoa, N., Mullis, S.W., Csinos, A.S. & Webster, T.M. First Report of Yellow Nutsedge (*Cyperus esculentus*) and Purple Nutsedge (*C. rotundus*) in Georgia Naturally Infected with *Impatiens necrotic spot virus*. *Plant Dis*. **88**, 771–771 (2004).

Martinez-Zubiaur, Y., Chang Sidorchuk, L., González Alvarez, H., Barboza Vargas, N. & González Arias, G. First Molecular Evidence of *Tomato chlorotic spot virus* Infecting Tomatoes in Cuba. *Plant Dis*. **100**, 1956–1956 (2016).

Martinez, D.L.O., Zavaleta-Mejía, E., Mora-Aguilera, G. & Johansen N., R.M. Implications of weed composition and thrips species for the epidemiology of tomato spotted wilt in chrysanthemum ( Dendranthema grandiflora ). *Plant Pathol*. **48**, 707–717 (1999).

Martínez, R.T., Poojari, S., Tolin, S.A., Cayetano, X. & Naidu, R.A. First Report of *Tomato spotted wilt virus* in Peppers and Tomato in the Dominican Republic. *Plant Dis*. **98**, 163–163 (2014).

Marys, E. *et al.* The First Report of *Tomato spotted wilt virus* on Gerbera and Chrysanthemun in Venezuela. *Plant Dis*. **98**, 1161–1161 (2014).

Massumi, H., Samei, A., Pour, A.H., Shaabanian, M. & Rahimian, H. Occurrence, Distribution, and Relative Incidence of Seven Viruses Infecting Greenhouse-Grown Cucurbits in Iran. *Plant Dis*. **91**, 159–163 (2007).

Massumi, H., Shaabanian, M., Pour, A.H., Heydarnejad, J. & Rahimian, H. Incidence of Viruses Infecting Tomato and Their Natural Hosts in the Southeast and Central Regions of Iran. *Plant Dis*. **93**, 67–72 (2009).

Materazzi, A. & Triolo, E. *Spathiphyllum* sp.: A New Natural Host of *Impatiens necrotic spot virus*. *Plant Dis*, 85, 448–448.

Matsuura, S., Kubota, K. & Okuda, M. (2007). First Report of *Chrysanthemum stem necrosis virus* on Chrysanthemums in Japan. *Plant Dis*. **91**, 468–468 (2001).

Matsuura, S., Shigeru, H., Hideaki, H., Tetsuyuki, K., Kyoji, H. & Omura, T. Effects of Latent Infection of Stock Plants and Abundance of Thrips on the Occurrence of *Tomato spotted wilt virus* in Chrysanthemum Fields. *J. Gen. Plant Pathol*. **68**, 99–102 (2002).

Mavric, I. & Ravnikar, M. First report of *Tomato spotted wilt virus* and *Impatiens necrotic spot virus* in Slovenia. *Plant Dis*. **85**, 1288 (2001).

McGovern, R.J., Polston, J.E. & Harbaugh, B.K. Detection of a Severe Isolate of Impatiens Necrotic Spot Virus Infecting Lisianthus in Florida. *Plant Dis*, 81, 1334–1334.

Mertelik, J., Mokra, V., Gotzova, B. & Gabrielova, S. (2000). First Report of *Impatiens necrotic spot virus* in the Czech Republic. *Plant Dis*. **84**, 1045–1045 (1997).

Momol, M. T., Olson, S. M., Funderburk, J. E., Stavisky, J. & Marois, J. J. Integrated Management of Tomato Spotted Wilt on Field-Grown Tomatoes. *Plant Dis*. **88**, 882–890 (2004).

Momol, M. T., Pappu, H. R., Dankers, W., Rich, J. R. & Olson, S. M. First Report of *Tomato spotted wilt virus* in Habanero and Tabasco Peppers in Florida. *Plant Dis*. **84**, 1154–1154 (2000).

Moore, E., & Andessen, E. E. Notes on Plant Virus Diseases in South Africa. I. The Kromnek Disease of Tobacco and Tomato. *Dept.Agric.,Union of South Africa, Sci.Bull.* **182**, 1–36 (1939).

Moore, E. The Kromnek or Kat River Diease of Tobacco and Tomato in the East Province (South Africa). *Dept.Agric.,Union of South Africa,Sci.Bull*. **123**, 5–28 (1933).

Morales, F.., Arroyave, J.A., Castillo, J. & Leon，C.D. Cytopathology of *Mazie chlorotic mottle virus* in *Zea mays* L. *Maydica*. **44**, 231–235 (1999).

Moreno, A. *et al.* The incidence and distribution of viruses infecting lettuce, cultivated Brassica and associated natural vegetation in Spain. *Ann. Appl. Biol*. **144**, 339–346 (2004).

Mortazavi, N. & Aleosfoor, M. Efficiency of Thrips tabaci and *Frankliniella occidentalis* populations in transmission of *Tomato yellow ring virus*. *Zool. Ecol*. **25**, 241–246 (2015).

Mortazavi, N., Aleosfoor, M. & Minaei, K. Transmission of cineraria isolate of tomato yellow ring virus by *Frankliniella occidentalis* and Thrips tabaci (Thysanoptera , Thripidae). *Linzer Biol. beitrage J*. 2011–2018 (2013).

Moussa, A. Ben, Makni, M., Marrakchi, M. & Moussa, A. Ben. Identification of the principal viruses infecting tomato crops in Tunisia. *EPPO Bull*. 293–296 (2000).

Ben Moussa, A., Marrakchi, M. & Makni, M. Characterisation of Tospovirus in vegetable crops in Tunisia. *Infect Genet Evol*. **5**, 312–322 (2005).

Mullis, S.W., Csinos, A.S., Gitaitis, R.D. & Martinez-Ochoa, N. First Report of Pinaceae in Georgia Naturally Infected with *Tomato spotted wilt virus*. *Plant Dis*. **90**, 376–376 (2006).

Mullis, S.W. *et al.* First Report of Vidalia Onion (*Allium cepa*) Naturally Infected with *Tomato spotted wilt virus* and *Iris yellow spot virus* (Family *Bunyaviridae*, Genus *Tospovirus* ) in Georgia. *Plant Dis*. **88**, 1285–1285 (2004).

Mumford, R.A., Barker, I. & Wood, K.R. The Biology of the Tospoviruses. *Ann. Appl. Biol*. **128**, 159–183 (1996).

Mumford, R.A., Jarvis, B., Morris, J. & Blockley, A. First report of *Chrysanthemum stem necrosis virus* (CSNV) in the UK. *Plant Pathol*. **52**, 779–779 (2003).

Nacional, V., Plata, D. La, Plata, N.D. La, Celular, B. & Brasilia, U. De. Characterization of the N Gene of a Groundnut Ringspot Virus (GRSV) Isolate from Argentina. *J Phytopathol*. **717**, 713–717 (1995).

Nagata, T., Almeida, A. C. L., Resende, R. O. & de Avila, A. C. The competence of four thrips species to transmit and replicate four tospoviruses. *Plant Pathol*. **53**, 136–140 (2004).

Nagata, T., Inoue-Nagata, A.K., van Lent, J., Goldbach, R. & Peters, D. Factors determining vector competence and specificity for transmission of *Tomato spotted wilt virus*. *J. Gen. Virol*. **83**, 663–71 (2002b).

Nagata, T., Inoue-Nagata, A.K., Prins, M., Goldbach, R. & Peters, D. Impeded Thrips Transmission of Defective *Tomato spotted wilt virus* Isolates. *Phytopathol.* **90**, 454–459 (2000).

Nagata, T., Inoue-Nagata, A.K., Smid, H.M., Goldbach, R. & Peters, D. Tissue tropism related to vector competence of *Frankliniella occidentalis* for tomato spotted wilt tospovirus. *J Gen. Virol*. **80** ( Pt 2), 507–15 (1999).

Nagata, T., Resende, R. de O., Kitajima, E.W., Costa, H., Inoue-Nagata, A.K. & de Ávila, A.C. First Report of Natural Occurrence of Zucchini Lethal Chlorosis Tospovirus on Cucumber and Chrysanthemum Stem Necrosis Tospovirus on Tomato in Brazil. *Plant Dis*. **82**, 1403–1403 (1998).

Naidu, R.A., Deom, C.M. & Sherwood, J.L. First report of *Frankliniella fusca* as a vector of *Impatiens necrotic spot tospovirus*. *Plant Dis*. **85**, 1211 (2001).

Niblett, C. & Clafin, L. Maize lethal necrosis-a new virus disease of maize in Kansas. *Plant Dis. Report* **62**, 15–19 (1978).

Nikolić, D. *et al.* First Report of *Tomato spotted wilt virus* on *Brugmansia* sp. in Serbia. *Plant Dis*. **97**, 850–850 (2013).

Nischwitz, C., Mullis, S. W., Gitaitis, R. D. & Csinos, A. S. First Report of *Tomato spotted wilt virus* in Soybean (*Glycine max*) in Georgia. *Plant Dis*. **90**, 524–524 (2006a).

Nischwitz, C., Mullis, S. W., Gitaitis, R. D., Csinos, A. S. & Olson, S. M. First Report of *Tomato spotted wilt virus* in Leek (*Allium porrum*) in the United States. *Plant Dis*. 90, 525–525 (2006b).

Ogada, P. A., Maiss, E. & Poehling, H. M. Influence of tomato spotted wilt virus on performance and behaviour of western flower thrips (*Frankliniella occidentalis*). *J. Appl. Entomol*. **137**, 488–498 (2013).

Okazaki, S. *et al.* The effect of virus titre on acquisition efficiency of *Tomato spotted wilt virus* by *Frankliniella occidentalis* and the effect of temperature on detectable period of the virus in dead bodies. *Australas Plant Pathol*. **40**, 120–125 (2011).

Okazaki, S., Okuda, M., Komi, K., Yoshimatsu, H. & Iwanami, T. Overwintering Viruliferous *Frankliniella occidentalis* (Thysanoptera: Thripidae) as an Infection Source of *Tomato spotted wilt virus* in Green Pepper Fields. *Plant Dis*. **91**, 842–846 (2007).

Okuda, M., Fuji, S., Okuda, S., Sako, K. & Iwanami, T. Evaluation of the potential of thirty two weed species as infection sources of *Impatiens necrotic spot virus*. *J Plant Pathol*. **92**, 357–361 (2010).

Okuda, S., Okuda, M., Matsuura, S., Okazaki, S. & Iwai, H. Competence of *Frankliniella occidentalis* and *Frankliniella intonsa* strains as vectors for *Chrysanthemum stem necrosis virus*. *Eur. J. Plant Pathol*. **136**, 355–362 (2013).

Olaya, N., Betancourt, M., Cuellar, W. & Pappu, H. R.. First Report of natural infection of tomato (*Solanum lycopersicum* L.) and bell pepper (*Capsicum annuum*) by *Alstroemeria necrotic streak virus* in Colombia . *Plant Dis*. **101(6)**, 1065 (2016).

Pappu, H. R., Jones, R. A. C. & Jain, R. K. Global status of tospovirus epidemics in diverse cropping systems: Successes achieved and challenges ahead. *Virus Res*. **141**, 219–236 (2009).

Pappu, S. S., Black, M. C., Pappu, H. R., Brenneman, T. B., Culbreath, A. K. & Todd, J. W. First Report of Natural Infection of Peanut (Groundnut) by Impatiens Necrotic Spot Tospovirus (Family *Bunyaviridae* ). *Plant Dis*. **83**, 966–966 (1999).

Parrella, G. First report of *Parietaria mottle virus* in *Mirabilis jalapa*. *Plant Pathol*. **51**, 401–401 (2002).

Parrella, G., Greco, B., Cavicchi, L. & Bellardi, M.G. Dieback and Wilting Caused by *Tomato spotted wilt virus* in *Arctotis* × *hybrida* in Italy. *Plant Dis*. **97**, 1387–1387 (2013).

Parrella, G., Greco, B. & Troiano, E. Severe Symptoms of Mosaic and Necrosis in Bell Pepper Associated With *Parietaria mottle virus* in Italy. *Plant Dis*. **100**, 1514–1514 (2016).

Parrella, G., Greco, B. & Troiano, E. First Report of *Parietaria mottle virus* Associated With Yellowing Disease in *Diplotaxis tenuifolia* in Italy. *Plant Dis.* **101**, 850 (2017).

Pérez-Colmenares, Y. *et al.* Identification of *Tomato spotted wilt virus* Associated With Fruit Damage During a Recent Virus Outbreak in Pepper in Venezuela. *Plant Dis*. **99**, 896–896 (2015).

Pietersen, G. & Morris, J. Natural Occurrence of *Groundnut ringspot virus* on Soybean in South Africa. *Plant Dis*. **86**, 1271–1271 (2002).

Polizzi, G. & Bellardi, M. G. First Report of *Tomato spotted wilt virus* on *Coprosma repens* (*Mirror Bush*) in Italy. *Plant Dis*. **91**, 1362–1362 (2007).

Pourrahim, R., Farzadfar, S., Moini, A. A., Shahraeen, N. & Ahoonmanesh, A. First Report of Tomato Spotted Wilt Virus on Potatoes in Iran. *Plant Dis*. **85**, 442–442 (2001).

Quito-Avila, D. F., Alvarez, R. A., and Mendoza, A. A. Occurrence of maize lethal necrosis in Ecuador: A disease without boundaries? *Eur. J. Plant Pathol.* **146**, 705-710 (2016).

Rabelo, L., Pedrazzoli, D., Novaes, Q., Nagata, T., Rezende, E. & Kitajima, J. High incidence of tomato chlorotic spot virus in the state of Sao Paulo, Brazil. *Fitopatol. Bras*. **27**, 105–112 (2002).

Ramasso, E., Roggero, P., Dellavalle, G. & Lisa, V. Necrosi apicale del pomodoro causata da un Ilarvirus. *Inf. Fitopatol*. **1**, 71–77 (1997).

Rasoulpour, R. & Izadpanah, K. Characterisation of cineraria strain of *Tomato yellow ring virus* from Iran. *Australas. Plant Pathol*. **36**, 286–294 (2007).

Renukadevi, P. *et al.* First Report of *Tomato spotted wilt virus* Infection of Chrysanthemum in India. *Plant Dis*. **99**, 1190–1190 (2015).

Rico, P. & Hernández, C. Infectivity of in vitro transcripts from a full-length cDNA clone of pelargonium flower break virus an experimental and a natural host. *J Plant Pathol*. (2006).

Rico, P., Hernandez, C. & Hernández, C. Complete nucleotide sequence and genome organization of *Pelargonium flower break virus*. *Arch. Virol*. **149**, 641–651 (2004).

Roca, E., Aramburu, J. & Moriones, E. Comparative host reactions and *Frankliniella occidentalis* transmission of different isolates of tomato spotted wilt tospovirus from Spain. *Plant Pathol*. **46**, 407–415 (1997).

Roggero, P. *et al.* Necrotic disease in tomatoes in Greece and southern Italy caused by the tomato strain of *Parietaria mottle virus.* *J. Plant Pathol*. **82**, 159 (2000).

Roggero, P., Masenga, V. & Tavella, L. Field Isolates of *Tomato spotted wilt virus* Overcoming Resistance in Pepper and Their Spread to Other Hosts in Italy. *Plant Dis*. **86**, 950–954 (2002).

Rosales, M., Pappu, H.., Arayam, C. & Aljaro, A. Characterization of *Tomato spotted wilt virus* (*Tospovirus*, *Bunyaviridae*) from lettuce (*Lactuca sativa*) in Chile. *Phytopathol.* **97**, S101 (2007).

Rotenberg, D. *et al.* Variation in *Tomato spotted wilt virus* Titer in *Frankliniella occidentalis* and Its Association with Frequency of Transmission. *Phytopathol.* **99**, 404–410 (2009).

Sakurai, T., Inoue, T. & Tsuda, S. Distinct efficiencies of Impatiens necrotic spot virus transmission by five thrips vector species (Thysanoptera: Thripidae) of tospoviruses in Japan. *Appl. Entomol. Zool*. 39, 71–78 (2004).

Salamon, P., Nemes, K., Salánki, K. & Palkovics, L. First Report of Natural Infection of Pea (*Pisum sativum*) by *Tomato spotted wilt virus* in Hungary. *Plant Dis*. **96**, 295–295 (2012).

Salvalaggio, A.E., López Lambertini, P.M., Cendoya, G. & Huarte, M.A. Temporal and spatial dynamics of *Tomato spotted wilt virus* and its vector in a potato crop in Argentina. *Ann. Appl. Biol*. **171**, 5–14 (2017).

Samuel, G., Bald, J. & Pittman, H. Investigations on “spotted wilt” of tomatoes. *Aust. Counc. Sci. Ind. Res. Bull*. **44**, 1–64 (1930).

Scott, S.W., Zimmerman, M.T. & Rankin, D. Complete sequence of the RNA 1 and RNA 2 of *Parietaria mottle virus*. *Arch. Virol*. **151**, 1895–1898 (2006).

Sevik, M.A. & Arli-Sokmen, M. Estimation of the effect of *Tomato spotted wilt virus* (TSWV) infection on some yield components of tomato. *Phytoparasitica* **40**, 87–93 (2012).

Shahraeen, N., Ghotbi, T. & Mehraban, A.H. Occurrence of *Impatiens necrotic spot virus* in Ornamentals in Mahallat and Tehran Provinces in Iran. *Plant Dis*. **86**, 694–694 (2002).

Shalileh, S., Ogada, P. A., Moualeu, D. P. & Poehling, H. M. Manipulation of *Frankliniella occidentalis* (Thysanoptera: Thripidae) by Tomato Spotted Wilt Virus (*Tospovirus*) Via the Host Plant Nutrients to Enhance Its Transmission and Spread. *Environ. Entomol*. **45**, 1235–1242 (2016).

Sherman, J.M., Moyer, J.W. & Daub, M.E. Tomato Spotted Wilt Virus Resistance in Chrysanthemum Expressing the Viral Nucleocapsid Gene. *Plant Dis*. **82**, 407–414 (1998).

Shrestha, A., Sundaraj, S., Culbreath, A.K., Riley, D.G., Abney, M.R. & Srinivasan, R. Effects of Thrips Density, Mode of Inoculation, and Plant Age on Tomato Spotted Wilt Virus Transmission in Peanut Plants. *Environ. Entomol*. **44**, 136–143 (2015).

Sin, S. H., McNulty, B. C., Kennedy, G. G. & Moyer, J. W. Viral genetic determinants for thrips transmission of *Tomato spotted wilt virus*. *Proc. Natl. Acad. Sci. U. S. A*. **102**, 5168–5173 (2005).

Spadotti, D., Leão, E., Rocha, K., Pavan, M.A. & Krause-Sakate, R. First report of *Groundnut ringspot virus* in cucumber fruits in Brazil. *New Dis. Reports* **29**, 25 (2014).

Stanković, I., Bulajić, A., Vučurović, A., Ristić, D., Jović, J. & Krstić, B. First Report of *Tomato spotted wilt virus* on *Gerbera hybrida* in Serbia. *Plant Dis*. **95**, 226–226 (2011).

Stanković, I. *et al.* First Report of *Tomato spotted wilt virus* Infecting Onion and Garlic in Serbia. *Plant Dis*. **96**, 918–918 (2012).

Stanković, I. *et al.* First Report of *Tomato spotted wilt virus* on Chrysanthemum in Serbia. *Plant Dis*. **97**, 150–150 (2013).

Stone, B. O. M. & Hollings, M. Some properties of pelargonium flower-break virus. *Ann. Appl. Biol*. **75**, 15–23 (1973).

Subbaiah, K. V., Sai Gopal, D. V. R. & Krishna Reddy, M. First Report of a Tospovirus on Sunflower (*Helianthus annus* L.) from India. *Plant Dis*. **84**, 1343–1343 (2000).

Testa, M., Sanna, D., Pintore, R., Marongiu, G. & Marras P. A. A two-year survey of TSWV occurrence in globe artichokes in Sardinia (Italy). Acta Hort. **917**, 297–302 (2011).

Teyssandier, E. E., DalBo, E. & Nome, S. F. Maize virus diseases in Argentina. In: *Proc. Int. Maize Virus Dis. Colloq. Workshop.* Ohio Agricultural Research and Development Center, Wooster, OH., pp. 93–99 (1981).

Tóth, E.K., Kriston, É., Takács, A., Bajtek, M., Kazinczi, G. & Horváth, J. First Report of *Impatiens necrotic spot virus* in Ornamental Plants in Hungary. *Plant Di.* **91**, 331–331 (2007).

Trkulja, V. *et al.* First Report of *Impatiens necrotic spot virus* on Begonia in Bosnia and Herzegovina. *Plant Dis*. **97**, 1004–1004 (2013a).

Trkulja, V. *et al.* First Report of *Tomato spotted wilt virus* on Gloxinia in Bosnia and Herzegovina. *Plant Dis*. **97**, 429–429 (2013b).

Vaira, A.M., Roggero, P., Luisoni, E., Masenga, V., Milne, R.G. & Lisa, V. Characterization of two Tospoviruses in Italy : tomato spotted wilt and impatiens necrotic spot. *Plant Pathol*. 530–542 (1993).

Vicchi, V., Fini, P. & Cardoni, M. Presence of impatiens necrotic spot tospovirus (INSV) on vegetable crops in Emilia-Romagna region. *Inf. Fitopatol*. **49**, 52–55 (1999).

Wangai, A. W., Mandal, B., Pappu, H. R. & Kilonzo, S. Outbreak of *Tomato spotted wilt virus* in Tomato in Kenya. *Plant Dis*. **85**, 1123–1123 (2001).

Wangai, A. W. *et al.* First Report of *Maize chlorotic mottle virus* and Maize Lethal Necrosis in Kenya. *Plant Dis*. **96**, 1582–1582 (2012).

Warfield, C. Y., Clemens, K. & Adkins, S. First Report of *Tomato chlorotic spot virus* on Annual Vinca (*Catharanthus roseus*) in the United States. *Plant Dis*. **99**, 895 (2015).

Webster, C.G. *et al.* Emergence of *Groundnut ringspot virus* and *Tomato chlorotic spot virus* in Vegetables in Florida and the Southeastern United States. *Phytopathol.* **105**, 388–398 (2015).

Webster, C. G., States, U., Pierce, F., Perry, K. L. & Lu, X. First Report of *Groundnut ringspot virus* Infecting Tomato in South Florida. *Plant Heal Prog*. 2–4 (2010).

Webster, C. G. & Turechek, W. W. Expansion of *Groundnut ringspot virus* Host and Geographic Ranges in Solanaceous Vegetables in Peninsular Florida. *Plant Heal Prog* (2011).

Wei, M. S., Li, G. F., Ma, J. & Kong, J. First Report of *Pelargonium flower break virus* Infecting *Pelargonium* Plants in China. *Plant Dis*. **99**, 735 (2015).

van de Wetering, F., van der Hoek, M., Goldbach, R., Mollema, C. & Peters, D. Variation in tospovirus transmission between populations of *Frankliniella occidentalis* (Thysanoptera: Thripidae). *Bull Entomol Res*. **89**, 579–588 (1999).

Whitfield, A. E., Campbell, L. R., Sherwood, J. L. & Ullman, D. E. Tissue Blot Immunoassay for Detection of *Tomato spotted wilt virus* in *Ranunculus asiaticus* and Other Ornamentals. *Plant Dis*. **87**, 618–622 (2003).

Wijkamp, I. Distinct Levels of Specificity in Thrips Transmission of Tospoviruses. *Phytopathol.* **85**, 1069 (1995).

Wijkamp, I., Goldbach, R. & Peters, D. Differential susceptibilities between leaf disks and plants in the transmission of tomato spotted wilt virus by *Frankliniella occidentalis* to TSWV hosts and transgenic plants. *J. Phytopathol. Zeitschrift* **144**, 355–362 (1996a).

Wijkamp, I., Goldbach, R. & Peters, D. Propagation of tomato spotted wilt virus in *Frankliniella occidentalis* does neither result in pathological effects nor in transovarial passage of the virus. *Entomol. Exp. Appl*. **81**, 285–292 (1996b).

Wijkamp, I. & Peters, D. Determination of the Median Latent Period of Two Tospoviruses in *Frankliniella occidentalis*, using a Novel Leaf Disc Assay. *Phytopathol.* **83**, 986–991 (1993).

Williams, L. V., Lambertini, P. M. L., Shohara, K. & Biderbost, E. B. Occurrence and Geographical Distribution of Tospovirus Species Infecting Tomato Crops in Argentina. *Plant Dis*. **85**, 1227–1229 (2001).

Wilson, C. R., Wilson, A. J. & Pethybridge, S. J. First Report of *Tomato spotted wilt virus* in Common Agapanthus. *Plant Dis*. **84**, 491–491 (2000).

Wu, K., Zheng, K.Y., Zhang, Z.K., McBeath, J.H. & Dong, J.H. First report of *Crinum asiaticum* as a natural host of tomato zonate spot virus in China. *J. Plant Pathol*. **97**, 76 (2016).

Wu, P. R. *et al.*. Genetic and serological characterization of chrysanthemum stem necrosis virus, a member of the genus *Tospovirus*. *Arch. Virol*. **160**, 529–536 (2015).

Xiao, L. *et al.* First Report of *Tomato spotted wilt virus* Infecting Cowpea in China. *Plant Dis*. **100**, 233–233 (2016).

Xie, L., Zhang, J., Wang, Q., Meng, C., Hong, J. & Zhou, X. P. Characterization of Maize Chlorotic Mottle Virus Associated with Maize Lethal Necrosis Disease in China. *J Phytopathol*. **159**, 191–193 (2011).

Yardımcı, N. & Kılıç, H.Ç. Tomato spotted wilt virus in vegetable growing areas in the west mediterranean region of Turkey. *African J. Biotechnol*. **8**, 4539–4541 (2009).

Yoon, J. Y., Choi, G. S. & Choi, S. K. First Report of *Chrysanthemum stem necrosis virus* on *Chrysanthemum morifolium* in Korea. *Plant Dis*. **101**, 264–264 (2017a).

Yoon, J. Y., Choi, G. S. & Choi, S. K. First Report of *Tomato spotted wilt virus* in *Eustoma grandiflorum* in Korea. *Plant Dis*. **101**, 515–515 (2017b).

Zarzyńska-Nowak, A., Rymelska, N., Borodynko, N. & Hasiów-Jaroszewska, B. The Occurrence of *Tomato yellow ring virus* on Tomato in Poland. *Plant Dis*. **100**, 234–234 (2016).

Zhang, Q., Ding, Y.M. & Li, M. First Report of *Impatiens necrotic spot virus* Infecting *Phalaenopsis* and *Dendrobium* Orchids in Yunnan Province, China. *Plant Dis*. **94**, 915–915 (2010).

Zhao, M., Ho, H., Wu, Y., He, Y. & Li, M. Western Flower Thrips (*Frankliniella occidentalis*) Transmits Maize Chlorotic Mottle Virus. *J. Phytopathol*. **162**, 532–536 (2014).

Zhao, W. *et al.* Effect of Spinosad Resistance on Transmission of Tomato Spotted Wilt Virus by the Western Flower Thrips (Thysanoptera: Thripidae). *J. Econ. Entomol*. **109**, 62–69 (2016).

Zheng, X., Zhang, J., Chen, Y., Dong, J. & Zhang, Z. Effects of Tomato Zonate Spot Virus infection on the development and reproduction of its vector *Frankliniella occidentalis* (Thysanoptera: Thripidae). *Florida Entomol*. **97**, 549–554 (2014).

Zheng, Y. X., Huang, C. H., Cheng, Y. H., Kuo, F. Y. & Jan, F. J. First Report of *Tomato spotted wilt virus* in Sweet Pepper in Taiwan. *Plant Dis*. **94**, 920–920 (2010).

Zindović, J., Bulajić, A., Krstić, B., Ciuffo, M., Margaria, P. & Turina, M. First Report of *Tomato spotted wilt virus* on Pepper in Montenegro. *Plant Dis*. **95**, 882–882 (2011).
